# Development and validation of two SCORE-based cardiovascular risk prediction models for Eastern Europe: a multicohort study

**DOI:** 10.1093/eurheartj/ehaa571

**Published:** 2020-07-04

**Authors:** Taavi Tillmann, Kristi Läll, Oliver Dukes, Giovanni Veronesi, Hynek Pikhart, Anne Peasey, Ruzena Kubinova, Magdalena Kozela, Andrzej Pajak, Yuri Nikitin, Sofia Malyutina, Andres Metspalu, Tõnu Esko, Krista Fischer, Mika Kivimäki, Martin Bobak

**Affiliations:** Department of Epidemiology & Public Health, University College London, 1-19 Torrington Place, London WC1E 7HB, UK; Centre for Non-Communicable Disease, Institute for Global Health, University College London, 30 Guilford Street, London WC1N 1EH, UK; Estonian Genome Center, Institute of Genomics, University of Tartu, Riia 23b, 51010 Tartu, Estonia; Department of Applied Mathematics Computer Science and Statistics, Ghent University, Krijgslaan 281, S9, 9000 Ghent, Belgium; Research Center in Epidemiology and Preventive Medicine, University of Insubria, Via O. Rossi 9, 21100 Varese, Italy; Department of Epidemiology & Public Health, University College London, 1-19 Torrington Place, London WC1E 7HB, UK; Department of Epidemiology & Public Health, University College London, 1-19 Torrington Place, London WC1E 7HB, UK; Centre for Environmental Health Monitoring, National Institute of Public Health, Šrobárova 48, 10042 Prague, Czech Republic; Department of Epidemiology and Population Studies, Institute of Public Health, Jagiellonian University Medical College, ul. Grzegórzecka 20, 31531 Krakow, Poland; Department of Epidemiology and Population Studies, Institute of Public Health, Jagiellonian University Medical College, ul. Grzegórzecka 20, 31531 Krakow, Poland; Research Institute of Internal and Preventive Medicine, Branch of the Institute of Cytology and Genetics, SB RAS, 10 Ac. Lavrentieva ave, 630090 Novosibirsk, Russia; Research Institute of Internal and Preventive Medicine, Branch of the Institute of Cytology and Genetics, SB RAS, 10 Ac. Lavrentieva ave, 630090 Novosibirsk, Russia; Novosibirsk State Medical University, Krasny Prospect 52, 630091 Novosibirsk, Russia; Estonian Genome Center, Institute of Genomics, University of Tartu, Riia 23b, 51010 Tartu, Estonia; Institute of Cell and Molecular Biology, University of Tartu, Riia 23b, 51010 Tartu, Estonia; Estonian Genome Center, Institute of Genomics, University of Tartu, Riia 23b, 51010 Tartu, Estonia; Estonian Genome Center, Institute of Genomics, University of Tartu, Riia 23b, 51010 Tartu, Estonia; Institute of Mathematics and Statistics, University of Tartu, Narva mnt 18, 51009 Tartu, Estonia; Department of Epidemiology & Public Health, University College London, 1-19 Torrington Place, London WC1E 7HB, UK; Department of Epidemiology & Public Health, University College London, 1-19 Torrington Place, London WC1E 7HB, UK

**Keywords:** Psychosocial deprivation, Socioeconomic factors, Cardiovascular diseases, Risk prediction, Sensitivity and specificity, Eastern Europe

## Abstract

**Aims:**

Cardiovascular disease (CVD) risk prediction models are used in Western European countries, but less so in Eastern European countries where rates of CVD can be two to four times higher. We recalibrated the SCORE prediction model for three Eastern European countries and evaluated the impact of adding seven behavioural and psychosocial risk factors to the model.

**Methods and results:**

We developed and validated models using data from the prospective HAPIEE cohort study with 14 598 participants from Russia, Poland, and the Czech Republic (derivation cohort, median follow-up 7.2 years, 338 fatal CVD cases) and Estonian Biobank data with 4632 participants (validation cohort, median follow-up 8.3 years, 91 fatal CVD cases). The first model (recalibrated SCORE) used the same risk factors as in the SCORE model. The second model (HAPIEE SCORE) added education, employment, marital status, depression, body mass index, physical inactivity, and antihypertensive use. Discrimination of the original SCORE model (*C*-statistic 0.78 in the derivation and 0.83 in the validation cohorts) was improved in recalibrated SCORE (0.82 and 0.85) and HAPIEE SCORE (0.84 and 0.87) models. After dichotomizing risk at the clinically meaningful threshold of 5%, and when comparing the final HAPIEE SCORE model against the original SCORE model, the net reclassification improvement was 0.07 [95% confidence interval (CI) 0.02–0.11] in the derivation cohort and 0.14 (95% CI 0.04–0.25) in the validation cohort.

**Conclusion:**

Our recalibrated SCORE may be more appropriate than the conventional SCORE for some Eastern European populations. The addition of seven quick, non-invasive, and cheap predictors further improved prediction accuracy.

## Graphical Abstract

**Graphical Abstract:**
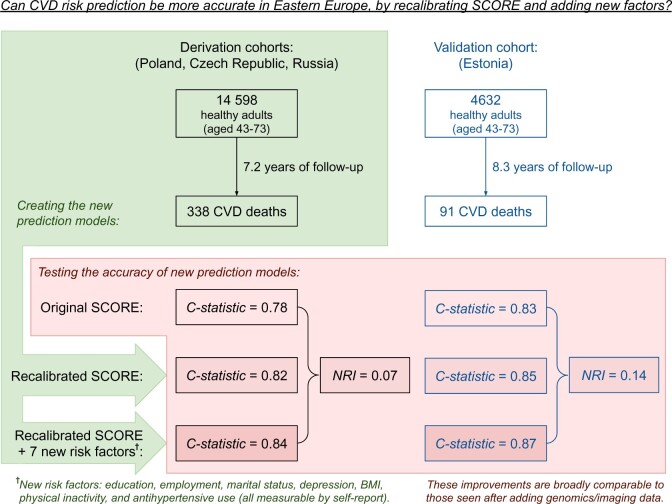

**See page 3334 for the editorial comment on this article (doi: 10.1093/eurheartj/ehaa685)**

## Introduction

The highest rates of cardiovascular disease (CVD) in the world are found in Eastern Europe. Age-standardized death rates of CVD (expressed as per 100 000, using 2013 data from the WHO European Mortality Indicator Database) are three to four times higher in Russia (547) when compared to the UK (141), Finland (187), or Germany (200). This calls for an urgent need to strengthen primary prevention in this region. An important aspect of primary prevention is risk stratification, which is commonly performed with computer-based prediction models to assess total CVD risk in healthy individuals. Accurate prediction enables behavioural and medical interventions, such as healthy lifestyle promotion (e.g. smoking cessation) and preventive lipid-lowering, antihypertensive, or anticoagulation medications to be targeted to those of highest risk.^1^ This agenda can reduce overtreatment and side effects for those at lower risk, while maximizing timely interventions, financial, health and equity gains for those at high risk. However, current risk prediction models in Eastern Europe remain far from perfect.

A major drawback is that there are few freely available models calibrated for Eastern European countries. The SCORE model, derived from 98% Western European participants,^2^ remains the default choice in Eastern Europe without recalibration (a method that adapts risk algorithm to account for differences in baseline risk between populations). At least one multi-centre analysis has demonstrated that SCORE is poorly calibrated for contemporary Eastern European populations, with three to eight predicted events for every one observed event.^3^ Although some countries like Poland have made nationally calibrated models, such country-specific models are unavailable for the majority of Eastern European countries. One solution is to recalibrate the original SCORE model, so it better suits the profile of Eastern European countries today.

To address this, we aimed to derive and externally validate two new risk models for the Eastern European region. First, we evaluated the performance of the conventional SCORE model and recalibrated this prediction tool, so that the risk coefficients and baseline hazard rates were optimized for contemporary cohort data from Eastern European countries. This procedure allows to minimize systematic under- and overestimation of risk, thereby giving more accurate estimates of absolute risk. Second, we tested whether adding self-reported information, which is cheap and easy to measure, would further improve risk stratification based on the recalibrated SCORE. Our focus was on psychosocial variables (e.g. depression, marital status, unemployment) that have previously been targets of risk prediction research but have not been validated in this geographical context.^4–14^ Finally, we validated the two modified models in an external cohort.

## Methods

A summary of the methods and results is shown in the *Graphical abstract*.

### Derivation data

To develop the new models, we used data from the population-based HAPIEE cohort study.^15^ Baseline data, including CVD risk factors, were collected between 2002 and 2008 from 34 873 individuals aged 43–73 years, from the Czech Republic, Poland, Russia, and Lithuania. Trained nurses performed a personal interview, physical examination and took blood samples. Serum cholesterol was determined by the automated enzymatic method. Past medical and drug history, education, employment, marital status, and physical inactivity were assessed by interview according to standardized questionnaire. Depressive symptoms were assessed using the Center for Epidemiologic Studies Depression Scale-20 (CESD-20) questionnaire (range 0–60), which was analytically dichotomized if CESD-20 ≥16.

**Figure 1:**
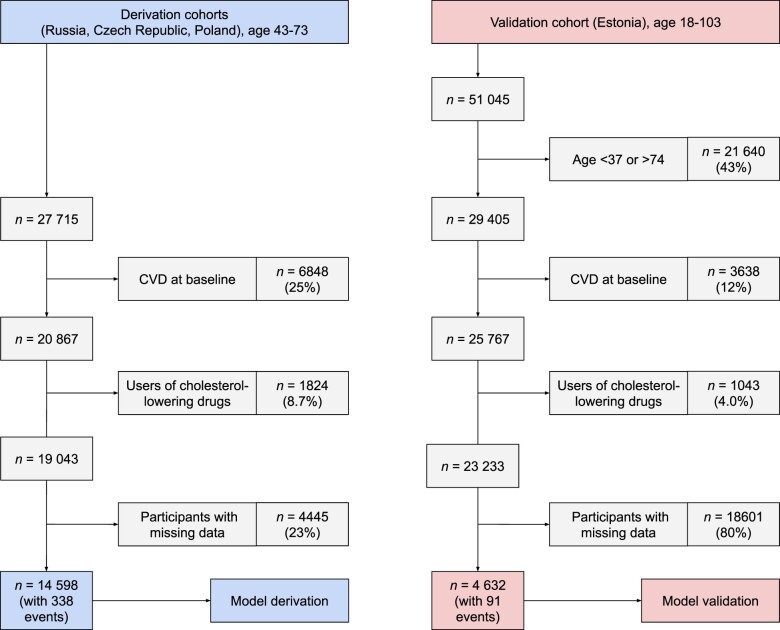
Flowchart illustrating participant selection into the derivation and validation datasets. CVD, cardiovascular disease.

We did not include the Lithuanian cohort, due to later entry into the study, and slight differences in data collection. We excluded participants who self-reported a diagnosis or previous hospitalization for angina, myocardial infarction, or stroke, those scoring positive on the Rose Angina questionnaire, and those taking lipid-lowering medication (as statins are thought to be a key intervention for high-risk people^2^). Twenty-three percent of participants had missing data on at least one of the variables and were excluded, resulting in 14 598 participants in the derivation sample (*Figure 1*).

As in the original SCORE model, only fatal outcomes were considered. Due to international variability in coding detailed causes of death, our primary outcome was *any CVD mortality* (ICD-10: I00–I99). Linkage to mortality registries in Russia, Poland, and Czech Republic (for a total of 8.0, 8.9, and 11.3 years) gave median follow-up times of 6.6, 7.1, and 9.6 years, respectively. Patients who did not develop outcomes were censored at 10 years, or earlier if their follow-up was shorter than 10 years. A total of 102 female and 236 male CVD deaths occurred in the derivation sample.

### External validation data

In the Estonian Biobank study, baseline data were collected between 2002 and 2011 from 51 045 population-based participants (median recruitment = 4 November 2008), typically by nurse practitioners at the patients’ primary care centre.^16^ Data collection was similar to the derivation data; however, validation data also included rural participants, a composite invitation method, and more data linkage for determining past medical history (Supplementary material online, *Methods S1*). Depression was assessed by a single-item with three response options ‘I do not have anxiety/depression’ (reference), vs. ‘I have moderate anxiety/depression’ or ‘I have severe anxiety/depression’ (these two options were merged in the analysis).

All participants who were eligible for our analysis (*n* = 23 233) provided blood samples for freezing and consented to additional analyses being performed in the future should this become possible. Subsequently, funding became available to measure plasma cholesterol by Proton nuclear magnetic resonance (NMR) spectroscopy (Brainshake Ltd) for a random sample of 4632 (22% of the original cohort) participants. The detailed rationale and methods for this sub-study are available elsewhere.^17^ In brief, as this subsample was selected at random, and since all selected samples were successfully thawed and analysed, we had little reason to believe that these participants were systematically different to those without measured cholesterol. The median and maximum follow-up time was 8.3 and 13.7, respectively. The final analytical sample had 4632 participants, of whom 91 died of CVD.

### Model development

Full details of model development are given in Supplementary material online, *Methods S2*. In brief, we fitted a single model for men and women across all countries in Eastern Europe. Each participant was allocated a binary dummy variable to denote whether they lived in a country with lower or higher baseline risk. We fitted three models. First, we used the original SCORE model.^5^ Second, we created ‘recalibrated SCORE’, a model with the same risk factors as in SCORE, but recalibrated using our derivation data. In this model, we also optimized how the existing SCORE risk factors were modelled. Accordingly, we used three categories for smoking (as opposed to two categories in SCORE) and added a cholesterol-squared term.

In the third step, we created the ‘HAPIEE SCORE’ model, by adding new risk factors that were available in both our derivation and validation cohorts. Each risk factor had to be easily measurable via self-report and needed previous meta-analytic evidence of association with CVD from population-based studies. Seven risk factors were added: body mass index (BMI) (weight in kg/height in m^2^, modelled with a linear term plus a squared term centred at 23 kg/m^2^), physical inactivity (<150 min per week), educational attainment (tertiary secondary or primary, modelled with equal risk differences between these categories), employment status (employed, unemployed, retired), marital status (single, widowed/divorced, married/cohabiting), possible depression (≥16 on CESD-20 questionnaire), and the use of antihypertensive medications and its interaction with systolic blood pressure.

### Calibration and discrimination

*Calibration* plots were used to determine the degree of over/underestimation of risk prediction among participants grouped into six risk strata. The number of strata was determined by the dual consideration of >10 events per strata and clinical meaningfulness of the threshold, to result in six strata using the following thresholds: 2.5%, 5%, 7.5%, 10%, and 20% absolute risk of CVD in the next 10 years. *Discrimination* was assessed using Harrell’s *C*-statistic, which extends the dichotomous receiver operating characteristic curves to survival models.^18^

### Net reclassification improvement

We dichotomized individuals at the 5% risk threshold for CVD mortality since the *European Society of Cardiology* guidelines suggest that this is the most important threshold to influence decisions on interventions like statins.^1^ *Net reclassification improvement* (*NRI*) is one way of measuring the extent to which a new model may outperform an older model in terms of sensitivity and specificity. It evaluates movement in four domains, by adding together the percentage of cases correctly predicted by only the new model, minus the percentage of cases incorrectly predicted by only the new model (*change in sensitivity*), plus the percentage of non-cases correctly predicted by only the new model, minus the percentage of non-cases incorrectly predicted by only the new model (*change in specificity*). The *Continuous NRI* assumes an infinite number of clinically meaningful risk thresholds, which we consider to be less clinically useful than the more conservative *Categorical NRI*. Improvements in *Categorical NRI* can be larger, if more categories are specified. We conservatively specified only one low-risk category and one high-risk category (divided at the 5% threshold for absolute risk of CVD death).

As a sensitivity analysis, *Reclassification Plots* were used to visually inspect reclassification performance across the entire risk spectrum (not just at across the 5% absolute risk boundary).

### Statistical analysis

Two Cox proportional hazards models were fitted (one for recalibrated SCORE and the other for HAPIEE SCORE) to estimate the association between each risk factor, and the time to CVD death. We used 1000 bootstraps to calculate 95% confidence intervals. Detailed R packages are given in Supplementary material online, *Methods S2*.

## Results

Descriptive statistics of the derivation and validation datasets are shown in Supplementary material online, *Table S1* and *Figure S2*. Briefly, the derivation dataset was three times larger than the validation dataset, which methodologically might be appropriate. Compared to the derivation cohort, the validation cohort had a more favourable CVD risk profile in some domains (e.g. age, sex, smoking, blood pressure, employment), while unfavourable in others (e.g. depression, single marital status, physical inactivity).

**Table 1:** Discrimination performance of three cardiovascular prediction models, as measured by Harrell’s C-statistic

	Name of model		
	Original SCORE	Recalibrated SCORE	HAPIEE SCORE
Derivation data			
C-statistic (95% CI)	0.783 (0.735–0.831)	0.818 (0.774–0.862)	0.840 (0.800–0.880)
Change in C-statistic		0.035	0.022
			0.057
Validation data			
C-statistic (95% CI)	0.832 (0.769–0.896)	0.851 (0.791–0.910)	0.865 (0.806–0.923)
Change in C-statistic		0.019	0.014
			0.033

CI, confidence interval.

### Calibration and discrimination

The regression coefficients for the newly derived models are shown in Supplementary material online, *Table S2*. Overall, these are comparable to those reported by other epidemiological cohort studies and prediction models. The baseline risk and the beta coefficient for age capture much of what we currently do not understand about CVD risk. As expected, the addition of seven new risk factors attenuated both these parameters.

**Table 2:** Changes to reclassification across three cardiovascular prediction models, in the derivation cohort

From model 1 (original SCORE) to model 2 (recalibrated SCORE)								
Status after follow-up	Predicted 10-year risk (original SCORE)	Predicted 10-year risk (recalibrated SCORE)		Reclassified		Net correctly reclassified		
		<5%	>5%	Increased risk	Decreased risk			
Died from CVD (*n* = 338)								
	<5%	67	10	3%		−11%		
	>5%	47	214		14%			
Did not die from CVD (*n* = 14 260)								
	<5%	8885	288	2%		15%		
	>5%	2377	2710		17%			
Categorical net reclassification improvement (95% CI)						0.03	(−0.01 to 0.08)	*P* = 0.09
Continuous net reclassification improvement (95% CI)						0.19	(0.08 to 0.29)	*P* < 0.0001

From model 2 (recalibrated SCORE) to model 3 (HAPIEE SCORE)								
Status after follow-up	Predicted 10-year risk (recalibrated SCORE)	Predicted 10-year risk (HAPIEE SCORE)		Reclassified		Net correctly reclassified		
		<5%	>5%	Increased risk	Decreased risk			
Died from CVD (*n* = 338)								
	<5%	90	24	7%		2.4%		
	>5%	16	208		5%			
Did not die from CVD (*n* = 14 260)								
	<5%	10 671	591	4%		0.4%		
	>5%	654	2344		5%			
Categorical net reclassification improvement (95% CI)						0.03	(−0.01 to 0.07)	*P* = 0.14
Continuous net reclassification improvement (95% CI)						0.44	(0.33 to 0.55)	*P* < 0.0001

From model 1 (original SCORE) to model 3 (HAPIEE SCORE)								
Status after follow-up	Predicted 10-year risk (original SCORE)	Predicted 10-year risk (HAPIEE SCORE)		Reclassified		Net correctly reclassified		
		<5%	>5%	Increased risk	Decreased risk			
Died from CVD (*n* = 338)								
	<5%	60	17	5%		−8.6%		
	>5%	46	215		14%			
Did not die from CVD (*n* = 14 260)								
	<5%	8774	399	3%		15%		
	>5%	2551	2536		18%			
Categorical net reclassification improvement (95% CI)						0.07	(0.02 to 0.11)	*P* = 0.005
Continuous net reclassification improvement (95% CI)						0.45	(0.34 to 0.56)	*P* < 0.0001

CI, confidence interval; CVD, cardiovascular disease.

Detailed calibration plots are shown in Supplementary material online, *Figure S4*. Calibration was near perfect (i.e. linear) in derivation data. In validation data, calibration was good among people with low, moderate, and high risks (absolute risk between 0% and 10%), which is where decisions on preventive interventions are typically made. Our new models overestimated risk among people of very high risk (i.e. absolute risk >10%), but this is unlikely to alter clinical management, as all these participants are eligible for statin therapy according to both the old and new prediction models.

In comparison with the original SCORE model, the recalibrated SCORE improved discrimination (change in *C*-statistic = +0.035 and +0.019 in derivation and validation cohorts, *Table 1*). The subsequent addition of seven new risk factors to the algorithm led to further improvements in *C*-statistic (+0.022 and +0.014). Our dataset was not well powered to explore the relative contribution of each of these seven variables, although in Supplementary material online, *Table S3* we report some preliminary exploratory analyses. Briefly, each new predictor seemed to make an additive contribution to improving discrimination performance. For comparison, when excluding three well-established biomedical risk factors (blood pressure, cholesterol, and diabetes status) from the final model in the derivation data, the decrease in discrimination (−0.022) was exactly the same as the decrease in discrimination seen when alternatively excluding the seven new variables (−0.022).

**Table 3:** Changes to reclassification across three cardiovascular prediction models, in the validation cohort

From model 1 (original SCORE) to model 2 (recalibrated SCORE)								
Status after follow-up	Predicted 10-year risk (original SCORE)	Predicted 10-year risk (recalibrated SCORE)		Reclassified		Net correctly reclassified		
		<5%	>5%	Increased risk	Decreased risk			
Died from CVD (*n* = 91)								
	<5%	27	11	12%		11%		
	>5%	1	52		1%			
Did not die from CVD (*n* = 4541)								
	<5%	3572	204	4%		−2.3%		
	>5%	98	667		2%			
Categorical net reclassification improvement (95% CI)						0.09	(0.02 to 0.16)	*P* = 0.02
Continuous net reclassification improvement (95% CI)						−0.09	(−0.28 to 0.10)	*P* = 0.34

From model 2 (recalibrated SCORE) to model 3 (HAPIEE SCORE)								
Status after follow-up	Predicted 10-year risk (recalibrated SCORE)	Predicted 10-year risk (HAPIEE SCORE)		Reclassified		Net correctly reclassified		
		<5%	>5%	Increased risk	Decreased risk			
Died from CVD (*n* = 91)								
	<5%	15	13	14%		12%		
	>5%	2	61		2%			
Did not die from CVD (*n* = 4541)								
	<5%	3309	361	8%		−6.4%		
	>5%	70	801		2%			
Categorical net reclassification improvement (95% CI)						0.06	(−0.02 to 0.14)	*P* = 0.16
Continuous net reclassification improvement (95% CI)						0.53	(0.37 to 0.68)	*P* < 0.0001

From model 1 (original SCORE) to model 3 (HAPIEE SCORE)								
Status after follow-up	Predicted 10-year risk (original SCORE)	Predicted 10-year risk (HAPIEE SCORE)		Reclassified		Net correctly reclassified		
		<5%	>5%	Increased risk	Decreased risk			
Died from CVD (*n* = 91)								
	<5%	14	24	26%		23%		
	>5%	3	50		3%			
Did not die from CVD (*n* = 4541)								
	<5%	3296	480	11%		−8.7%		
	>5%	83	682		2%			
Categorical net reclassification improvement (95% CI)						0.14	(0.04 to 0.25)	*P* = 0.006
Continuous net reclassification improvement (95% CI)						0.13	(−0.03 to 0.28)	*P* = 0.12

CI, confidence interval; CVD, cardiovascular disease.

Altogether, the two steps of first recalibrating and then adding seven extra risk factors demonstrated a substantial improvement in *C*-statistic, when comparing the final HAPIEE SCORE to the original SCORE model (+0.057 in derivation data and +0.033 in validation data).

### Net reclassification improvement

When comparing the original SCORE against the final HAPIEE SCORE, Categorical NRI was +0.07 (*P* = 0.005) in derivation data (*Table 2*) and +0.14 (*P* = 0.006) in validation data (*Table 3*). In the derivation data, NRI was driven by improvements in specificity. In the validation data, NRI was driven by improvements in sensitivity.

Our data had limited statistical power to explore to what extent this improvement was driven by recalibration or adding new risk factors. It seemed that both changes played a role, potentially with recalibration of the original model causing slightly larger improvements to Categorical NRI (+0.03 and +0.09 in derivation and validation data), when compared to the benefit of adding seven new risk factors to the recalibrated model (+0.03 and +0.06, respectively).

### Sensitivity analyses

We performed sensitivity analyses using two subgroups of participants to examine in greater detail potential benefits of adding the seven risk factors to the algorithm. First, when restricting the analyses to around one-quarter of intermediate-risk participants (whose absolute risk was between 2.5% and 7.5%), the *Categorical NRI* was twice as large (+0.06 and +0.17 in derivation and validation data). Second, when restricting the analyses to participants aged >60 years (of all risk profiles), the *Categorical NRI* was also larger (+0.07 and +0.23).

Although we find the *Continuous NRI* measure to be less clinically relevant, we also report these in *Tables 2* and *3* as this is common in the literature. As predicted, *Continuous NRI* was typically larger, ranging from +0.13 to +0.53 for 5 of the 6 model comparisons made. None of the three comparisons made in the derivation data were likely to have occurred by chance (*P* < 0.0001 in all three case). In the validation data, the improvement seen after adding new risk factors was unlikely a chance finding (*P* < 0.0001), but the recalibration step was the only one to result in a negative *Continuous NRI* (−0.09) and this could have happened by chance alone (*P* = 0.34). Reclassification plots (Supplementary material online, *Figure S5*) suggested that improvements to reclassification were generally distributed across the wider spectrum of risk, potentially with slightly larger benefits to those at intermediate risk.

In multiple imputation analyses to handle missing data (Supplementary material online, *Methods S3*), model discrimination and reclassification were little changed, when compared to analyses using complete case data (Supplementary material online, *Tables S5–S7*).

## Discussion

This study recalibrated the original SCORE model and also generated and validated a new prediction model including seven new risk factors that are measurable using self-report. Each of these two revisions to the original SCORE model led to large improvements to discrimination, in both the derivation and validation datasets. When comparing our final model (the HAPIEE SCORE) to the original SCORE, reclassification improved across the entire risk spectrum, as well as at a commonly used interventional threshold of 5% absolute CVD risk. Reclassification benefits were largest in participants at intermediate risk. Our findings suggest that in terms of risk prediction, simple questionnaire measures on behavioural and psychosocial factors may be as informative as established biomedical risk factors (blood pressure, cholesterol, diabetes) or alternatively data across the entire genome. As self-report variables are cheap and convenient to collect, this supports their incorporation into risk prediction models, particularly in the absence of equally predictive biomarkers.

These findings have two implications for public health and clinical practice: first, our recalibrated SCORE algorithm may be more accurate than the original SCORE algorithm, for default clinical use in some Eastern European countries. Second, the *European Society of Cardiologists*’ guidelines recommend ‘additional risk factor assessment if such a risk factor improves risk classification [e.g. by calculation of a net reclassification index] and if the assessment is feasible in daily practice’.^2^ We found self-report variables to improve risk classification. More research is now warranted on *feasibility*—for example by trialling various processes for collecting self-report data in either opportunistic or population-based settings.

### Strengths and limitations

An important strength of our study was the use of an independent cohort from a different country for external validation of the two new risk prediction models. Furthermore, the two datasets differed in terms of study design, recruitment, and data collection. Although this is not always ideal for studying reproducibility of findings, demonstrating that different lines of research lead to the same conclusions supports the robustness of our study.^19^ Second, we focused our analysis on clinically meaningful changes across an important interventional threshold. We are only aware of two previous reports in the literature where *Categorical NRI* improved after adding a psychosocial factor (education^16^ or long working hours^20^).

Our study had several limitations. First, the validation dataset was of borderline sufficient size, to evaluate some performance measures, and we did not have enough power to fit gender-specific models. Second, like previous cohort studies, our cohorts were prone to response rate bias, which may make them healthier than the general populations they represent. This may lead to an underestimation of risk in the general population, and an underusage of potentially beneficial interventions. In addition, it is unclear how well our models generalize to people who were not included in our study, such as the very young or old, ethnic minorities, as well as likely performance in other countries. Third, although improvements were seen in both datasets, our derivation dataset showed larger improvements. This may be because the original SCORE model was better suited for our validation data (thereby elevating the baseline standard that we sought to improve). Alternatively, our final models may contain residual overfitting. Fourth, in sensitivity analyses, we used multiple imputation for participants who had missing data. This approach relies on the assumption that data are missing at random, which might not necessarily be the case. The missing data are therefore a limitation of the study, although there is no reason to suspect that this would have a major impact on the findings. Fifth, improvements in risk factors and treatment over time may decrease the baseline risk, meaning that our model may overpredict risk in the future. Sixth, just like the SCORE model, our algorithms were not developed to predict the risk of non-fatal CVD.

### Comparison with previous studies

Previous studies have recalibrated the SCORE model to various countries, particularly in Western Europe. Some countries such as England have derived prediction models from electronic health records collected in primary care (i.e. professionals who will use the model).^21^ However, recalibration has rarely been performed in Eastern European countries. When this has been done, it has often taken place in the private sector, or the full results have not been published in peer-reviewed English language journals. Our study may be the first that updated SCORE for more than one Eastern European country and compared performance against the original SCORE model. The *C*-statistic from the external validation of our final model (0.87) is slightly better compared to external validation of other models that are clinically recommended (e.g. QRISK2 0.77–0.84^22^ and Pooled Cohorts Equation 0.65–0.72^23^ ^,^ ^24^), suggesting that our model may have clinical potential in Eastern Europe.

Previous studies that aimed to improve CVD risk prediction by adding new predictors have found this to be a difficult endeavour. Indeed, the *European Society of Cardiologists*’ guidelines warn how several emerging risk factors ‘may not have the ability to reclassify subjects’,^1^ highlighting that reclassification is insufficiently tested. Derivation studies have sometimes reported *C*-statistic improvements as large as 0.08,^25^ but the few studies also including external validation tend to report improvements up to 0.01 or 0.02.^26^ Our *C*-statistic improvement of 0.022 in the derivation and 0.014 in the validation datasets is consistent with this. For comparison, Abraham *et al*.^27^ added 49 310 genetic variants associated with heart disease to the prediction algorithm. Improvements in *C*-statistic (0.011–0.017) and Continuous NRI (0.25–0.37) were both comparable to those in our study (*C*-statistic 0.014–0.022; Continuous NRI 0.44–0.53). When a more detailed history of smoking, diabetes, kidney disease, and rheumatoid arthritis was added to QRISK in forming QRISK2, the improvements in *C*-statistic were smaller (0.003–0.004).^21^ There are a variety of novel risk factors that could potentially improve risk prediction accuracy. We hope these will converge on globally similar instruments, by following European guidelines that emphasize the feasibility of data collection (including cost) along with their ability to reclassify patients.

There is limited literature about improving prediction models by adding behavioural or psychosocial data. When ASSIGN and QRISK were developed, these added area-level psychosocial status and BMI, as well as model recalibration.^28^ Head-to-head model comparisons did not disentangle what part of the improvement came from recalibration, and what part from novel risk factors. Studies on the latter question were initially underpowered resulting in the risk of overfit.^4^ ^,^ ^5^ Better powered studies reported benefits smaller than what we report, as can be expected since we added not just one or two variables but seven.^6–13^ Analysis of Italian cohorts suggested that the addition of four risk factors (alcohol, occupational physical activity, sport physical activity, and job strain) improved prediction performance; however, this has not been externally validated.^14^ Altogether, our findings are consistent with the literature and extend this by demonstrating (potentially for the first time) the value of adding multiple behavioural and psychosocial variables in external data. Future research could derive new algorithms with similar risk factors, or alternatively evaluate the validity of our HAPIEE SCORE in other Eastern- or Western-European settings. We recognize that many clinicians prefer to base decision-making on laboratory measurements and imaging techniques rather than self-reported information. However, in resource-poor settings, validated risk prediction algorithms may offer a fast and cheap way of enhancing risk prediction, at least until other biomarkers become available.

## Conclusion and future research

Our findings provide clinicians and public health experts two validated SCORE-based risk prediction algorithms for Eastern Europe and further strengthen the argument that psychosocial factors can have real-life relevance. There is already a large evidence base to suggest that psychosocial risk factors (such as education, employment, marital status, employment, and depression) are strongly associated with CVD. Psychosocial factors can be upstream causes of health behaviours (such as smoking), causal effects more directly, or represent consequences of preclinical disease.^29^ ^,^ ^30^ If integrated into routine preventative practice, the assessment of psychosocial factors may additionally help to normalize public and clinician attitudes towards these variables as legitimate cardiovascular risk factors alongside smoking, blood pressure, and lipids.

Multiple other risk factors have been identified by cohort studies (such as dietary factors) that are largely missing from the risk prediction literature. We encourage others to attempt similar translational work in the future. There may be benefit in conceptualizing risk prediction as a multi-stage process, where factors already known in public data systems are analysed first (e.g. detailed medical history, including depression, smoking, marital status, and unemployment). In the second stage, some patients are invited to provide additional self-reported behavioural and psychosocial data (e.g. education, BMI, physical activity), sometimes using online tools. In the third stages, some patients may be invited for more expensive clinical investigations, such as blood pressure, cholesterol, ‘omics, and/or imaging. This agenda, if rigorously evaluated, may potentially create a more convenient, acceptable, effective and cost-effective cardiovascular screening programme for healthcare systems.

## Data availability

Data are available from the corresponding author upon request, after completing appropriate ethical approval.

## Funding

This work was supported by the Wellcome Trust [106554/Z/14/Z to T.T., 064947/Z/01/Z, and WT081081]; the National Institute for Health Research [Academic Clinical Lectureship to T.T.]; Medical Research Council [S011676 to M.K.]; the National Institute on Aging [1R01 and AG23522]; the National Institutes of Health [R01AG056477]; Research Foundation Flanders [1S05916N to O.D.]; Ghent University Special Research Fund [BOF.01P08419 to O.D.]; NordForsk; Academy of Finland [311492]; the Russian Scientific Foundation [14-45-00030 and 20-15-00371]; the the Russian Academy of Science [AAAA-A17-117112850280-2]; the National Science Centre of Poland [2018/29/B/NZ7/02118]; the Estonian Research Council [IUT20-60, PUT1660, and PUT1665]; the University of Tartu [SP1GVARENG]; and the European Union’s Regional Development Fund [2014-2020.4.01.15-0012 ‘GENTRANSMED’].

**Conflict of interest:** none declared.

## Supplementary Material

ehaa571_Supplementary_Data

## References

[1] Piepoli MF, Hoes AW, Agewall S, Albus C, Brotons C, Catapano AL, Cooney M-T, Corrà U, Cosyns B, Deaton C, Graham I, Hall MS, Hobbs FDR, Løchen M-L, Löllgen H, Marques-Vidal P, Perk J, Prescott E, Redon J, Richter DJ, Sattar N, Smulders Y, Tiberi M, van der Worp HB, van Dis I, Verschuren WMM. 2016 European Guidelines on cardiovascular disease prevention in clinical practice: the Sixth Joint Task Force of the European Society of Cardiology and Other Societies on Cardiovascular Disease Prevention in Clinical Practice. Eur Heart J 2016;37:2315–2381. 27222591 10.1093/eurheartj/ehw106 PMC4986030

[2] Conroy RM, Pyörälä K, Fitzgerald AE, Sans S, Menotti A, De Backer G, De Bacquer D, Ducimetiere P, Jousilahti P, Keil U, Njølstad I. Estimation of ten-year risk of fatal cardiovascular disease in Europe: the SCORE project. Eur Heart J 2003;24:987–1003. 12788299 10.1016/s0195-668x(03)00114-3

[3] Vikhireva O, Pajak A, Broda G, Malyutina S, Tamosiunas A, Kubinova R, Simonova G, Skodova Z, Bobak M, Pikhart H. SCORE performance in Central and Eastern Europe and former Soviet Union: MONICA and HAPIEE results. Eur Heart J 2014;35:571–577. 23786858 10.1093/eurheartj/eht189 PMC3938861

[4] Fiscella K, Tancredi D, Franks P. Adding psychosocial status to Framingham scoring to reduce disparities in coronary risk assessment. Am Heart J 2009;157:988–994. 19464408 10.1016/j.ahj.2009.03.019

[5] Vikhireva O, Broda G, Kubinova R, Malyutina S, Pająk A, Tamosiunas A, Skodova Z, Simonova G, Bobak M, Pikhart H. Does inclusion of education and marital status improve SCORE performance in Central and Eastern Europe and former Soviet Union? Findings from MONICA and HAPIEE cohorts. PLoS One 2014;9:e94344. 24714549 10.1371/journal.pone.0094344 PMC3979770

[6] Ramsay SE, Morris RW, Whincup PH, Papacosta AO, Thomas MC, Wannamethee SG. Prediction of coronary heart disease risk by Framingham and SCORE risk assessments varies by psychosocial position: results from a study in British men. Eur J Cardiovasc Prev Rehabil 2011;18:186–193. 21450664 10.1177/1741826710389394

[7] Ingle L, Carroll S, Stamatakis E, Hamer M. Benefit of adding lifestyle-related risk factors for prediction of cardiovascular death among cardiac patients. Int J Cardiol 2013;163:196–200. 21700349 10.1016/j.ijcard.2011.06.001

[8] Pujades-Rodriguez M, Timmis A, Stogiannis D, Rapsomaniki E, Denaxas S, Shah A, Feder G, Kivimaki M, Hemingway H. Psychosocial deprivation and the incidence of 12 cardiovascular diseases in 1.9 million women and men: implications for risk prediction and prevention. PLoS One 2014;9:e104671. 25144739 10.1371/journal.pone.0104671 PMC4140710

[9] Ferrario MM, Veronesi G, Chambless LE, Tunstall-Pedoe H, Kuulasmaa K, Salomaa V, Borglykke A, Hart N, Söderberg S, Cesana G, Jørgensen T; for the MORGAM Project. The contribution of educational class in improving accuracy of cardiovascular risk prediction across European regions: the MORGAM Project Cohort Component. Heart 2014;100:1179–1187. 24794139 10.1136/heartjnl-2013-304664

[10] Schnohr P, Marott JL, Kristensen TS, Gyntelberg F, Gronbaek M, Lange P, Jensen MT, Jensen GB, Prescott E. Ranking of psychosocial and traditional risk factors by importance for coronary heart disease: the Copenhagen City Heart Study. Eur Heart J 2015;36:1385–1393. 25681607 10.1093/eurheartj/ehv027

[11] Veronesi G, Gianfagna F, Giampaoli S, Chambless LE, Mancia G, Cesana G, Ferrario MM. Improving long-term prediction of first cardiovascular event: the contribution of family history of coronary heart disease and social status. Prev Med 2014;64:75–80. 24732715 10.1016/j.ypmed.2014.04.007

[12] Graversen P, Abildstrøm SZ, Jespersen L, Borglykke A, Prescott E. Cardiovascular risk prediction: can Systematic Coronary Risk Evaluation (SCORE) be improved by adding simple risk markers? Results from the Copenhagen City Heart Study. Eur J Prev Cardiol 2016;23:1546–1556. 26976846 10.1177/2047487316638201

[13] Colantonio LD, Richman JS, Carson AP, Lloyd‐Jones DM, Howard G, Deng L, Howard VJ, Safford MM, Muntner P, Goff DC. Performance of the atherosclerotic cardiovascular disease pooled cohort risk equations by social deprivation status. J Am Heart Assoc 2017;6:e005676. 28314800 10.1161/JAHA.117.005676 PMC5524046

[14] Veronesi G, Borchini R, Landsbergis P, Iacoviello L, Gianfagna F, Tayoun P, Grassi G, Cesana G, Ferrario MM; The Cohorts Collaborative Study in Northern Italy (CCSNI) Research Group. Cardiovascular disease prevention at the workplace: assessing the prognostic value of lifestyle risk factors and job-related conditions. Int J Pub Health 2018;63:723–732. 29802415 10.1007/s00038-018-1118-2 PMC6015612

[15] Peasey A, Bobak M, Kubinova R, Malyutina S, Pajak A, Tamosiunas A, Pikhart H, Nicholson A, Marmot M. Determinants of cardiovascular disease and other non-communicable diseases in Central and Eastern Europe: rationale and design of the HAPIEE study. BMC Pub Health 2006;6:255. 17049075 10.1186/1471-2458-6-255 PMC1626086

[16] Leitsalu L, Haller T, Esko T, Tammesoo M-L, Alavere H, Snieder H, Perola M, Ng PC, Mägi R, Milani L, Fischer K, Metspalu A. Cohort profile: Estonian Biobank of the Estonian Genome Center, University of Tartu. Int J Epidemiol 2015;44:1137–1147. 24518929 10.1093/ije/dyt268

[17] Fischer K, Kettunen J, Würtz P, Haller T, Havulinna AS, Kangas AJ, Soininen P, Esko T, Tammesoo M-L, Mägi R, Smit S, Palotie A, Ripatti S, Salomaa V, Ala-Korpela M, Perola M, Metspalu A. Biomarker profiling by nuclear magnetic resonance spectroscopy for the prediction of all-cause mortality: an observational study of 17,345 persons. PLoS Med 2014;11:e1001606. 24586121 10.1371/journal.pmed.1001606 PMC3934819

[18] Harrell FE, Lee KL, Mark DB. Tutorial in biostatistics multivariable prognostic models: issues in developing models, evaluating assumptions and adequacy, and measuring and reducing errors. Stat Med 1996;15:361–387. 8668867 10.1002/(SICI)1097-0258(19960229)15:4<361::AID-SIM168>3.0.CO;2-4

[19] Munafo MR, Davey-Smoth G. Robust research needs many lines of evidence. Nature 2018;553:399–401. 10.1038/d41586-018-01023-3 29368721

[20] KivimäKi M, Batty GD, Hamer M, Ferrie JE, Vahtera J, Virtanen M, Marmot MG, Singh-Manoux A, Shipley MJ. Using additional information on working hours to predict coronary heart disease: a cohort study. Ann Intern Med 2011;154:457–463. 21464347 10.1059/0003-4819-154-7-201104050-00003 PMC3151554

[21] Hippisley-Cox J, Coupland C, Vinogradova Y, Robson J, Minhas R, Sheikh A, Brindle P. Predicting cardiovascular risk in England and Wales: prospective derivation and validation of QRISK2. BMJ 2008;336:1475–1482. 18573856 10.1136/bmj.39609.449676.25 PMC2440904

[22] Collins GS, Altman DG. Predicting the 10 year risk of cardiovascular disease in the United Kingdom: independent and external validation of an updated version of QRISK2. BMJ 2012;344:e4181. 22723603 10.1136/bmj.e4181 PMC3380799

[23] Muntner P, Colantonio LD, Cushman M, Goff DC, Howard G, Howard VJ, Kissela B, Levitan EB, Lloyd-Jones DM, Safford MM. Validation of the atherosclerotic cardiovascular disease pooled cohort risk equations. JAMA 2014;311:1406–1415. 24682252 10.1001/jama.2014.2630 PMC4189930

[24] Emdin CA, Khera AV, Natarajan P, Klarin D, Baber U, Mehran R, Rader DJ, Fuster V, Kathiresan S. Evaluation of the pooled cohort equations for prediction of cardiovascular risk in a contemporary prospective cohort. Am J Cardiol 2017;119:881–885. 28061997 10.1016/j.amjcard.2016.11.042

[25] Ruwanpathirana T, Owen A, Reid CM. Review on cardiovascular risk prediction. Cardiovasc Ther 2015;33:62–70. 25758853 10.1111/1755-5922.12110

[26] Sattar N, Welsh P, Sarwar N, Danesh J, Di Angelantonio E, Gudnason V, Davey Smith G, Ebrahim S, Lawlor DA. NT-proBNP is associated with coronary heart disease risk in healthy older women but fails to enhance prediction beyond established risk factors: results from the British Women's Heart and Health Study. Atherosclerosis 2010;209:295–299. 19815210 10.1016/j.atherosclerosis.2009.09.016

[27] Abraham G, Havulinna AS, Bhalala OG, Byars SG, De Livera AM, Yetukuri L, Tikkanen E, Perola M, Schunkert H, Sijbrands EJ, Palotie A, Samani NJ, Salomaa V, Ripatti S, Inouye M. Genomic prediction of coronary heart disease. Eur Heart J 2016;37:3267–3278. 27655226 10.1093/eurheartj/ehw450 PMC5146693

[28] Woodward M, Brindle P, Tunstall-Pedoe H; for the SIGN Group on Risk Estimation. Adding social deprivation and family history to cardiovascular risk assessment: the ASSIGN score from the Scottish Heart Health Extended Cohort (SHHEC). Heart 2005;93:172–176. 10.1136/hrt.2006.108167 PMC1861393 17090561

[29] Carter AR, Gill D, Davies NM, Taylor AE, Tillmann T, Vaucher J, Wootton RE, Munafò MR, Hemani G, Malik R, Seshadri S, Woo D, Burgess S, Davey Smith G, Holmes MV, Tzoulaki I, Howe LD, Dehghan A. Understanding the consequences of education inequality on cardiovascular disease: Mendelian randomisation study. BMJ 2019;365:l1855. 31122926 10.1136/bmj.l1855 PMC6529852

[30] Kivimäki M, Steptoe A. Effects of stress on the development and progression of cardiovascular disease. Nat Rev Cardiol 2018;15:215–229. 29213140 10.1038/nrcardio.2017.189

