# Erratum to: Development and validation of two SCORE-based cardiovascular risk prediction models for Eastern Europe: a multicohort study

**DOI:** 10.1093/eurheartj/ehaa875

**Published:** 2020-10-09

[Eur Heart J 2020; doi:10.1093/eurheartj/ehaa571]

Upon the original publication of this article, several errors were noted. The publisher apologises for the following errors that have subsequently been corrected in the online and print versions of the article:

The affiliation footnote ‘7’ should read: “Department of Epidemiology and Population Studies, Institute of Public Health, Jagiellonian University Medical College, ul. Grzegoórzecka 20, 31531 Krakow, Poland”.

The “Graphical Abstract” figure should be replaced with the corrected version.

The “Take home” figure should be deleted.

In the “Methods” section, the following text should read: “A summary of the methods and results is shown in the Graphical abstract.”. In addition, two other corrections under the following headings were made:

Under “Derivation data”, the following sentence should read: “Trained nurses performed a personal interview, physical examination and took blood samples. Serum cholesterol was determined by the automated enzymatic method. Past medical and drug history, education, employment, marital status, and physical inactivity were assessed by interview according to standardized questionnaire.”;

Under “External validation data”, the following text should read: “between 2002 and 2011 from 51 045 population-based participants”.

The results in Table 1. should be corrected.

In Table 2., the “Categorical net reclassification improvement (95% CI)” and “Continuous net reclassification improvement (95% CI)” results for “From model 1 (original SCORE) to model 2 (recalibrated SCORE)”, “From model 2 (recalibrated SCORE) to model 3 (HAPIEE SCORE)” and “From model 1 (original SCORE) to model 3 (HAPIEE SCORE)” should be corrected. The results heading for “model 1 (original SCORE) to model 3 (HAPIEE SCORE)” should be: “Predicted 10-year risk (HAPIEE)”.

In Table 3., the “Categorical net reclassification improvement (95% CI)” and “Continuous net reclassification improvement (95% CI)” results for “From model 1 (original SCORE) to model 2 (recalibrated SCORE)”, “From model 2 (recalibrated SCORE) to model 3 (HAPIEE SCORE)” and “From model 1 (original SCORE) to model 3 (HAPIEE SCORE)” should be corrected. The results heading for “model 1 (original SCORE) to model 3 (HAPIEE SCORE)” should be: “Predicted 10-year risk (HAPIEE)”.

The funding section omitted the following: “the National Science Centre of Poland [2018/29/B/NZ7/02118]”.

In addition, two further corrections have been made to the online version of the article. These are as follows:

The affiliation footnote ‘11’ should read: “Institute of Mathematics and Statistics, University of Tartu, Narva mnt 18, 51009 Tartu, Estonia”.

The funding section omitted the following: “Research Foundation Flanders [1S05916N to O.D.]; Ghent University Special Research Fund [BOF.01P08419 to O.D.]”.

