# Supplementary material for: Development and validation of two SCORE-based cardiovascular risk prediction models for Eastern Europe: a multicohort study
Source: Eur Heart J. 2020 Jul 4;41(35):3325–33. doi: 10.1093/eurheartj/ehaa571 (PMC7544536; doi:10.1093/eurheartj/ehaa571)
Supplement: ehaa571_Supplementary_Data [file ehaa571_supplementary_data.zip › ehaa571_Supplementary_Data/Supplementary Material.docx]

Supplementary Materials for:

**Development and validation of two SCORE-based cardiovascular risk prediction models for Eastern Europe: a multicohort study**

**Contents** Page

Supplementary Methods 1 Data collection 2

Supplementary Methods 2 Model development 3

Supplementary Figure 1 Selecting the age band 6

Supplementary Figure 2 Age distribution 7

Supplementary Table 1 Baseline descriptive characteristics 8

Supplementary Table 1 Hazard Ratios of the new models 9

Supplementary Figure 3 Hazard functions for cholesterol 10

Supplementary Figure 4 Calibration 11

Supplementary Table 3 Sensitivity analysis: adding each factor one-by-one 12

Supplementary Table 4 Sensitivity analysis: deriving and validating on pooled data 13

Supplementary Figure 5 Reclassification 14

Supplementary Methods 3 Multiple imputation – Methods 15

Multiple imputation – Results 17

Supplementary Table 5 Multiple imputation – Discrimination 17

Supplementary Table 6 Multiple imputation – NRI, derivation dataset 18

Supplementary Table 7 Multiple imputation – NRI, derivation dataset 19

**Supplementary Methods 1 - Data collection**

**Recruitment and baseline data in the validation cohort (Estonia)**

Similarly to the derivation cohort, most data were collected using computer-assisted personal interviews and physical examinations. However, the validation cohort has a different sampling frame (urban-rural vs urban only in derivation cohort); different invitation method (opportunistic recruitment by family and hospital physicians, plus self-referral following a nationwide publicity campaign, vs postal invite only in derivation). The Estonia Biobank study also used the following additional sources to ascertain past medical and drug history (a) the participant’s primary care record; b) linkage with the national acute myocardial infarction registry, and c) a national repository of electronic healthcare records and prescriptions [covering approximately 80% of inpatient discharge letters and 80% of all dispensed prescriptions by Dec 2011]).

**Ascertainment of cardiovascular death outcomes**

I] Derivation cohorts

For participants from Novosibirsk, Russia, national registries of death were not available for research, and so deaths were ascertained from multiple sources. First, the register of mortality which was established during the MONICA project (that was initiated and managed at the same institute which hosts the co-authors of the current study). This dataset uses data from local death certificates, as per a protocol specified in the municipal Department of Civil Registration of Death Acts of Novosibirsk city (ZAGS). Second, cross-referencing with information from the local register of myocardial infarction and Stroke (again held at the same institute) and with case record data from the Regional Bureau of Medico Legal Expertise. Third, repeated waves of the study (using postal questionnaire and health examinations) identified some additional fatalities. Attempts were made to verify their ICD codes via the above sources, and to confirm death using the Address Bureau.

Any potential uncertainty with regards to the underlying cause of death (from per ICD codes) was manually investigated, to ensure full concordance between routine classification of deaths and the special mortality register. Previous analyses have shown that for any given year, discrepancy between the Institute’s mortality register and the official state data varied between 0.6% and 4.5% for all-cause deaths and from 0.5% to 1.6% for CVD deaths (Malyutina S, Bobak M, Simonova G, Nikitin Y, Bogatyrev S. Patterns of CVD mortality in post-Soviet countries after transition and estimates of balance between routine and register’s indicators. Conference paper: “Mortality in countries of the former Soviet Union. Fifteen years after breakup: change and continuity”, Kiev, 12-14 Oct 2006).

For participants in Poland, deaths were ascertained predominantly from population registers, with additional cross-referencing by postal surveys and direct contact with study participants. Causes of death was taken from death certificates. No further validation procedures were used.

For participants in the Czech Republic, deaths were ascertained from the national population register. Causes of death was taken from death certificates. No further validation procedures were used.

I] Validation cohort

For participants in Estonia, most information came from the Estonian Causes of Death Registry. This compiles data on all cases of death registered on Estonian territory and during Estonian foreign missions. Cause of death is determined by a medical doctor or a pathologist, which since 1997 has been coded using ICD-10. The Death certificate has three main sections – a) Direct causes b) Disease, injury or complications which caused the event in section a); and finally, section c) the Underlying cause of death that caused the event mentioned in b). If cardiovascular deaths were listed in any of these sections (a, b, or c) then this was defined as an event for the purposes of this study. No external validation is carried out of this registry.

**Supplementary Methods 2 - Model development**

**Choice of age band**

As the Estonian health system is developing prototypes for national CVD screening, we validated the newly-derived models as close as possible to eventual real-life settings. One key variable is the age range among whom to conduct screening. In countries like the UK, screening for CVD is for men aged >40 and women aged >50. Given higher baseline rates in countries like Estonia, this equates to an equivalent absolute level of risk as seen in men aged >37 and women aged >47 in Estonia (Supplementary Figure 1), which we took as the lower age limits for our validation study. It is unclear at what age any geriatric risks associated with statins exceed the cardiac benefits, but a commonly used upper threshold in other models is 74 years, which we also applied to Estonia.

**Data pooling**

The original SCORE project pooled data across various countries to create separate two models for “low risk” countries (Germany, France, Belgium, Italy, Spain), and “high risk” countries (Russia, Finland, Sweden, Norway, Denmark, UK). This original division could be best thought of as a Northern Europe (high risk) versus Southern Europe (low risk). Since then, the European Society of Cardiology has substantially added to and moved the categorization of each country. It has also moved some countries like Russia into a new third category, called “very-high risk countries”. However, an accompanying third model has not been derived for very-high risk countries, who are advised to use the regular high-risk model while *“taking into account that high-risk charts may underestimate risk in very-high risk countries”.* Overall, the majority of the countries that originally contributed to SCORE derivation are now no longer found in the same category where they were when SCORE was derived. Accordingly, we decided to discard country categorizations used by SCORE when deriving our two new models, and instead chose a new country categorization, to reflect the data we had access to.

There were not enough events in each country, so we divided the region into two halves. Cardiovascular mortality rates are higher in those Eastern European countries that were previously members of the Soviet Union (Estonia, Latvia, Lithuania, Belarus, Ukraine, Georgia, Russia) as well as those that border the Black Sea (Romania, Bulgaria). We considered cohorts from these countries to have a higher baseline risk, which was modelled with an additional individual-level categorical variable (“higher risk region”). This was compared against the reference category (“lower risk region”), which included participants from cohorts in Central European countries that border Germany and Austria. Due to limitations in statistical power, we made the assumption that this dichotomous variable would capture most of the heterogeneity in baseline risk between countries (that is, we did not fit random effects, a stratified Cox model, nor within-region country indicator variables).

The original SCORE project created separate models for men and women. While this can improve prediction performance, this requires sufficient events in each derivation arm. A conservative rule of thumb is to have at least 10 events per one coefficient in a newly derived model (with debate continuing around whether this should be relaxed^[[1]](#footnote-2)^). We had only 43 female events in the high-risk region (Russia). Such events are too few to reliably develop models with 10 to 20 coefficients in this subgroup, as this would render the models prone to spuriously fitting the coefficients too close to the derivation data, meaning that they are unlikely to work well in external validation data (a problem known as statistical overfit). To avoid this, we assumed that each risk factor (such as smoking) increases the Relative Risk of CVD for women just as much as it should for men, and it should have similar effects in both the low- and high-risk regions alike. However, since international differences CVD tend to be larger in men than women, we hypothesized that international differences in the baseline rate (among those with no CVD risk factors) should be larger in men than women. Accordingly, we fitted a single gender-by-area interaction term (male*high-risk-area). Overall, by making the assumptions that risk factors operated consistently across gender and country based on our prior knowledge, this allowed us to pool more individuals into a single model. By using more individuals in the model development phase, we expect our final models to perform better in future populations, such as our external validation cohort.

**Model Fitting**

Model 1 (*“Original SCORE”*)

We used the high-risk SCORE model, where two parametric Weibull models generated the baseline hazard function for men and women, respectively. Hazards increased in the presence of one of three risk factors (cholesterol > 6 mmol/L, modelled linearly; systolic blood pressure > 120 mmHg, modelled linearly; binary smoking status).

Model 2 (*“Recalibrated SCORE”*)

Cox regression was used to estimate a new baseline hazard and new coefficients for each of the SCORE risk factors, in a pooled dataset combining genders and both areas. Time-to event was used as the underlying time variable in the Cox model, which automatically adjusted for differences in entry time between participants (left truncation).

Our review of the literature suggested that additional benefit may be gained from more sophisticated modelling of existing predictors. Accordingly, we added an additional category of *Light smokers* (which combined *“Former Smokers”*, current smokers who *“Smoke < 1 cigarette per day”,* as well as smokers with a *“< 1 pack year history”)*.

We trialled quadratic functions to represent non-linear associations for cholesterol and/or age. An *a priori* criteria for keeping these was that when comparing nested models (using the *anova* command), the quadratic term should improve the P-value by >0.05. Age-squared had no benefit (p=0.36) and was dropped. Cholesterol-squared (centred at 6mmol/L) showed additional benefit (p=0.002, supplementary figure 3) and was kept. With 9 coefficients in the Recalibrated SCORE model, we had 34 events per each coefficient, denoting a small possibility for overfit.

Model 3 (*“HAPIEE SCORE”*)

After we had created the Recalibrated SCORE model, we tried to improve its performance by adding new risk factors to create HAPIEE SCORE. Candidate factors were selected as follows: Take all risk factors used in other CVD risk prediction models. Add further risk factors with meta-analytic evidence of independent associations with cardiovascular disease in conventional cohort studies. Eliminate those not measured in our derivation and validation cohorts. Eliminate markers that are impossible to detect using self-report (e.g. those requiring blood tests). Following this seven candidate predictors were identified. The HAPIEE SCORE model had 21 coefficients, meaning we fitted 16 events per each coefficient, which denotes a small possibility for overfit.

**Statistical analysis**

The programme R was used for all statistical analyses, along with its additional packages *survival, Hmisc, rms* and *mice*. Discrimination analyses were performed using the R commands *coxph* (for fitting)*, survfit* (for baseline risk) and *validate;* *rcorr.cens* (to validate each model, with 1000 bootstraps). We followed the advice of Harrell (who first proposed the C-statistic) to not perform hypothesis testing by inspecting P-values or the overlap between bootstrapped confidence intervals, when comparing two C-statistics from two models.^^[[2]](#footnote-3)^^

NRI was computed using the command reclassification from the R package “PredictABEL”. This uses the following formula:

Continuous NRI = P(up∣event)−P(down∣event) + P(down∣nonevent)−P(up∣nonevent)

Where “up” indicates the situation where individual’s risk estimate increases with the updated model while compared to old model and “down” indicated the situation where individual’s risk estimate decreases with the updated model while compared to the old model. So P(up∣event) is the proportion of events, whose risk estimates increase with updated model and P(down∣event) indicates the proportion of events whose risk estimates decrease with the updated model. In the similar manner for non-events, we observe the proportions of them who benefit or disadvantage from the updated model.

Categorical NRI is a more conservative subtype of Continuous NRI, which only counts movement (up or down) across a clinically prespecified categories (in our case, 5% risk of CVD), while discarding movements within low risk and movements within high risk categories. The hypothesis for continuous (as well as for categorical) NRI is:

H0: NRI=0

H1: NRI=!0

The authors of NRI have shown that simple asymptotic test for this hypothesis pair is based on z-statistic which follows a normal distribution (formula 9 from*^[[3]](#footnote-4)^*). This assumes independence between event and non-event individuals, and follows McNemar’s logic for signiﬁcance testing in correlated proportions. P-values are 2-sided. Analyses were done with R package “PredictABEL”.

External validation included the same discrimination, calibration, reclassification analyses for the derivation cohort.

**Supplementary Figure 1** Rates of CVD mortality, per 100 000 inhabitants. Data are from the Global Burden of Disease Study 2015^[[4]](#footnote-5)^, which relies on WHO Mortality data for the UK and Estonia. *Bold italic* font denotes age bands where screening for CVD has been suggested or attempted among Western European countries. Green arrows denote the corresponding age threshold in Estonia.
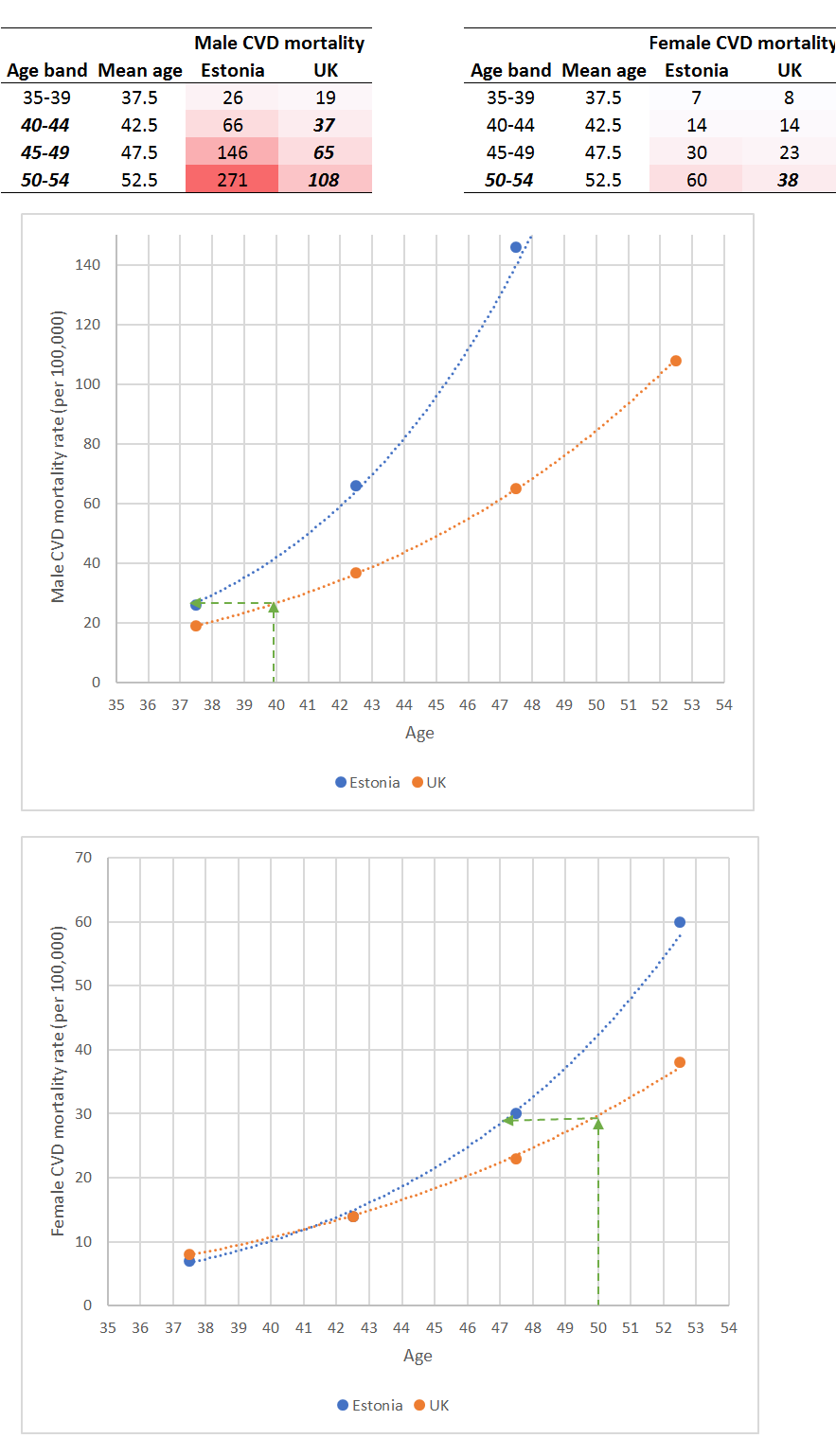


**Supplementary Figure 2** Age distribution of the validation and derivation datasets. The top pane shows the absolute frequency of participants in each age band. The bottom pane shows the relative frequency of participants in each age band.


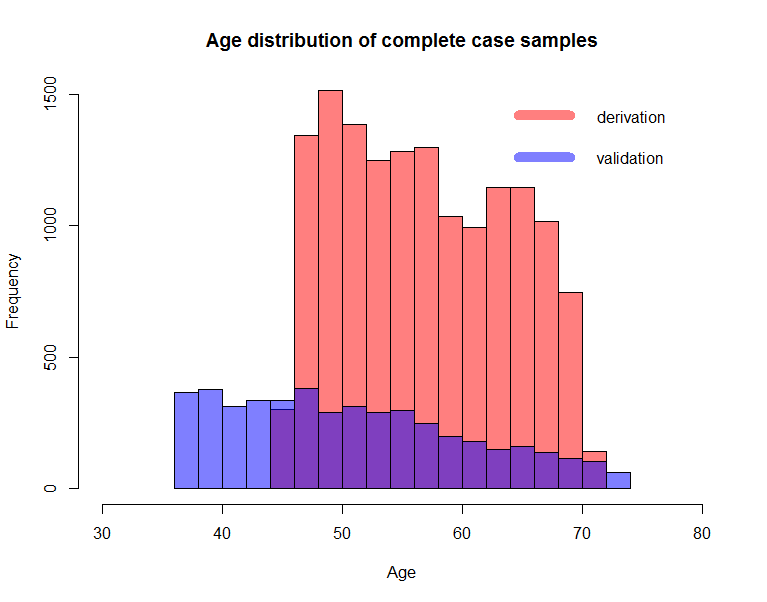


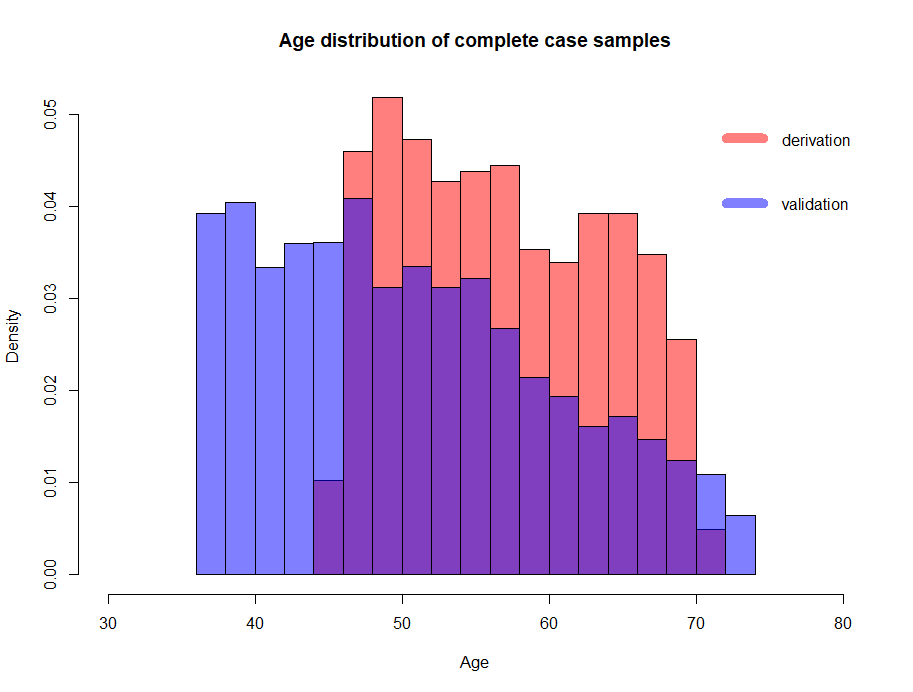


**Supplementary Table 1** Baseline characteristics of the analytical samples.

(Samples are complete cases, combined across two genders.)

| **Dataset** | **Derivation** | | **Validation** | |
| --- | --- | --- | --- | --- |
| ***Population characteristics*** |  | |  | |
| Country | Czech Republic,  Poland, and Russia | | Estonia | |
| Participants, N | 14,598 | | 4,632 | |
| Follow-up, median years | 7.2 | | 8.3 | |
| CVD mortality events (%) | 338 | (2.3%) | 91 | (2.0%) |
| ***SCORE risk factors*** |  | | | |
| Male, N (%) | 6,910 | (47%) | 1,563 | (34%) |
| Age, mean | 57 (range 44 to 72) | | 51 (range 37 to 74) | |
| Diabetes (%) | 953 | (6.5%) | 294 | (6.3%) |
| Smoking Status |  |  |  |  |
| never (%) | 6,853 | (47%) | 2,652 | (57%) |
| ex/light (%) | 3,615 | (25%) | 699 | (15%) |
| current (%) | 4,130 | (28%) | 1,281 | (28%) |
| Blood pressure, systolic (mmHg) | 139 | (± 22) | 129 | (± 16) |
| Total cholesterol (mmol/L) | 6.0 | (± 1.1) | 6.0 | (± 1.1) |
| ***Novel risk factors*** |  | | | |
| Body Mass Index (kg/m^2^) | 27.9 | (± 4.8) | 27.2 | (± 5.4) |
| Physically inactive (%) | 1,057 | (7.2%) | 2,480 | (54%) |
| Antihypertensive use | 3,805 | (26%) | 1,163 | (25%) |
| Education |  |  |  |  |
| tertiary (%) | 3,874 | (27%) | 1,147 | (25%) |
| secondary (%) | 9,509 | (65%) | 3,423 | (74%) |
| primary or less (%) | 1,215 | (8%) | 62 | (1.3%) |
| Employment Status: |  |  |  |  |
| Employed (%) | 8,425 | (58%) | 3,714 | (80%) |
| Unemployed (%) | 679 | (4.7%) | 102 | (2.2%) |
| Retired (%) | 5,494 | (38%) | 816 | (18%) |
| Marital Status: |  |  |  |  |
| Married/cohabiting (%) | 11,129 | (76%) | 2,544 | (55%) |
| Divorced/widowed (%) | 2,843 | (20%) | 1,586 | (34%) |
| Single (%) | 626 | (4.3%) | 502 | (11%) |
| Depression, suspected (%) | 2,979 | (20%) | 1,530 | (33%) |

**Supplementary Table 2** Hazard Ratios (also known as exponentiated beta coefficients) of two new models, from the derivation model. (The same parameters are also reported with greater precision (4 decimal places) in the file *“Supplementary calculator.xls”*).

|  |  | **Name of new model** | |
| --- | --- | --- | --- |
|  |  | **Recalibrated SCORE** | **HAPIEE SCORE** |
|  | Baseline risk | 0.25% | 0.09% |
| ***SCORE risk factors*** | |  |  |
|  | Male | 1.46 (1.05 to 2.02) | 1.94 (1.38 to 2.73) |
|  | Age (per 1 year) | 1.10 (1.08 to 1.12) | 1.09 (1.07 to 1.12) |
|  | Cholesterol, total (per 1 mmol/L) | 1.04 (0.93 to 1.14) | 1.06 (0.96 to 1.18) |
|  | Cholesterol^2^ (centred at 6 mmol/L) | 1.04 (1.02 to 1.06) | 1.04 (1.02 to 1.06) |
|  | Smoking status |  |  |
|  | Non smoker | reference | reference |
|  | Ex/light smoker | 1.59 (1.15 to 2.21) | 1.54 (1.11 to 2.14) |
|  | Current smoker | 3.48 (2.60 to 4.66) | 3.08 (2.28 to 4.16) |
|  | Blood pressure, systolic (per 1 mmHg) | 1.02 (1.01 to 1.02) | 1.02 (1.01 to 1.03) |
|  | Diabetes | 2.30 (1.68 to 3.16) | 2.23 (1.61 to 3.08) |
|  | High-risk area | 2.03 (1.33 to 3.11) | 1.80 (1.16 to 2.80) |
|  | High-risk area*Male interaction | 1.58 (0.97 to 2.58) | 1.96 (1.18 to 3.24) |
| ***Novel risk factors*** | |  |  |
|  | BMI (per 1-unit kg/m^2^) | - | 0.96 (0.92 to 1.00) |
|  | BMI^2^ (centered at 23 kg/m^2^) | - | 1.01 (1.00 to 1.01) |
|  | Physical Inactivity (<150 min./week) | - | 1.57 (1.14 to 2.18) |
|  | Antihypertensive use |  | 1.24 (0.77 to 2.00) |
|  | Antihypertensives*blood pressure interaction | | 0.99 (0.98 to 1.00) |
|  | Educational attainment^☨^ | - | 1.21 (1.00 to 1.47) |
|  | Employment Status | - |  |
|  | Employed | - | reference |
|  | Unemployed | - | 2.17 (1.32 to 3.56) |
|  | Retired | - | 1.39 (1.04 to 1.84) |
|  | Marital Status | - |  |
|  | Married/cohabiting | - | reference |
|  | Divorced/widowed | - | 1.48 (1.13 to 1.94) |
|  | Single | - | 2.66 (1.70 to 4.17) |
|  | Depression (possible) | - | 1.68 (1.30 to 2.16) |
|  |  |  |  |
| ^☨^ = for education, reference category was *"tertiary education or above"*. This was compared against two categories, assuming a linear fit across the three groups: *"secondary education"* and *"primary or less"*. | | | |
|  |  |  |  |
|  |  |  |  |

**Supplementary Figure 3** Shape of association between cholesterol and CVD death.

Rationale: In our main analysis, we showed that cholesterol-squared was a significant predictor of CVD risk. To further examine this in more detail, we explored below an alternative model that fitted a spline function (with 5 degrees of freedom). This allowed us to see whether a more complex shape might better describe the association between cholesterol and CVD in our datasets. Both models shown below were otherwise fitted as per the Recalibrated SCORE model, with the exception that the linear and squared cholesterol terms were replaced with a spline function.

Graphical interpretation: The spline shapes below are similar to a U shape. This suggests that a simple quadratic (cholesterol-squared) is appropriate, as using more parameters added limited additional value.


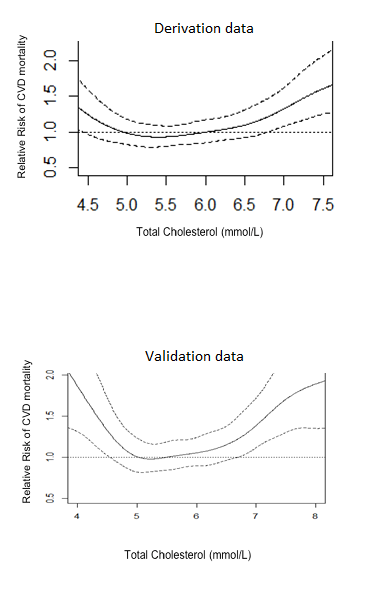
**Supplementary Figure 4** Calibration plots.


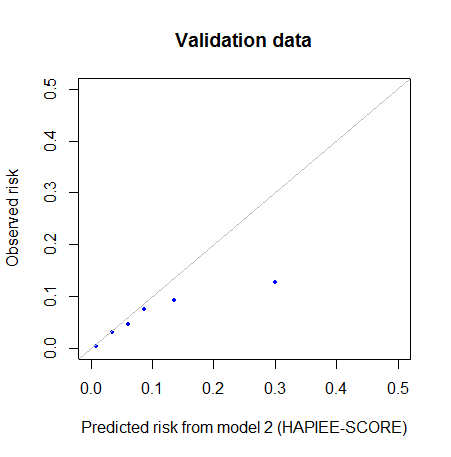

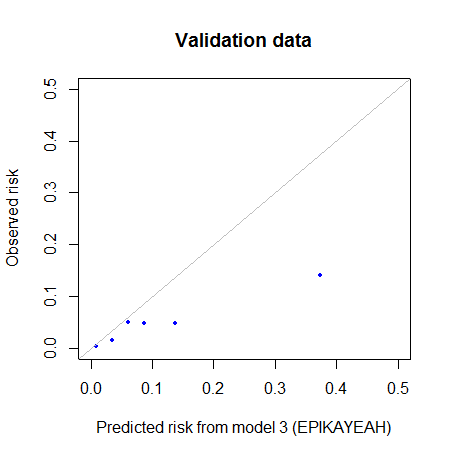

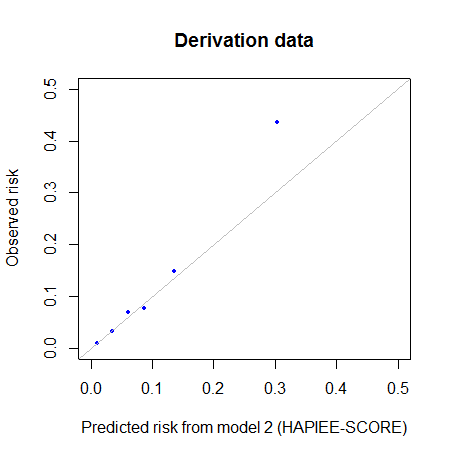

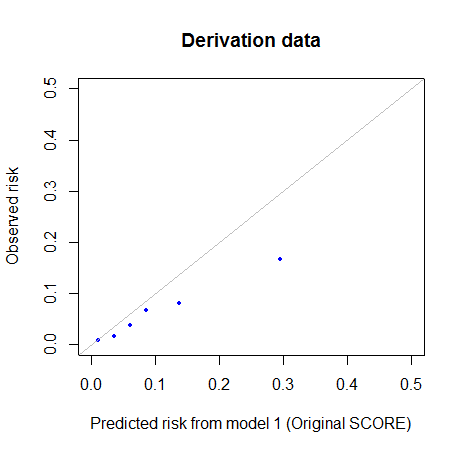

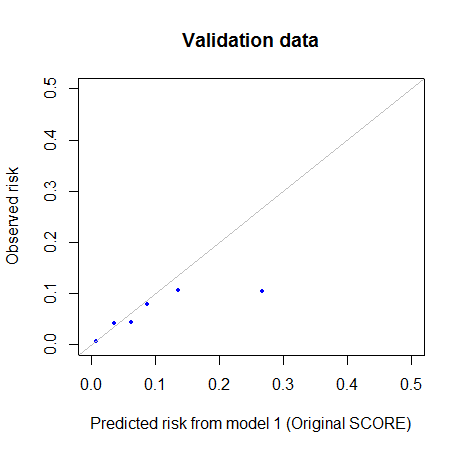

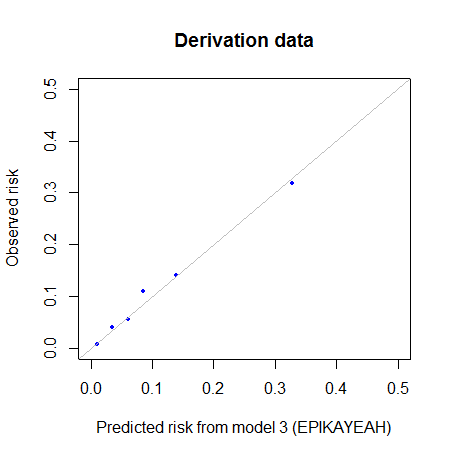


Predicted risk, using the Original SCORE model

Predicted risk, using the Original SCORE model

Predicted risk, using the Recalibrated SCORE model

Predicted risk, using the Recalibrated SCORE model

Predicted risk, using the final HAPIEE SCORE model

Predicted risk, using the final HAPIEE SCORE model

Predicted risk, using the Original SCORE model

**Supplementary Table 3** Exploratory analyses, investigating the increase in discrimination performance (as measured by Harrell’s C-Statistic), following the addition of single questions to the Reclassified SCORE model. All measures are taken from the derivation dataset.

| **Description of model** | **C-statistic** | **Change in C-statistic** |  |
| --- | --- | --- | --- |
| 1.Recalibrated SCORE | 0.818 | reference |  |
| 2.Recalibrated SCORE + Employment status | 0.824 | +0.006 |  |
| 3.Recalibrated SCORE + BMI + BMI^2^ | 0.823 | +0.005 |  |
| 4.Recalibrated SCORE + Depression | 0.822 | +0.004 | These seven components, when summed, total +0.027 |
| 5.Recalibrated SCORE + Physical Inactivity | 0.822 | +0.004 |  |
| 6.Recalibrated SCORE + Education | 0.821 | +0.003 |  |
| 7.Recalibrated SCORE + Marital Status | 0.821 | +0.003 |  |
| 8.Recalibrated SCORE + Antihypertensive use + Antihypertensive use*blood pressure | 0.820 | +0.002 |  |
| 9.Recalibrated SCORE + 4 psychosocial factors (i.e. + emp + dep + edu + mar) | 0.831 | +0.013 | These two, when summed, total +0.022 |
| 10.Recalibrated SCORE + 3 biobehavioural factors (i.e. + BMI + physinact + antihyp + antihyp*bp ) | 0.827 | +0.009 |  |
| 11.HAPIEE SCORE | 0.840 | +0.022 |  |

Interpretation: When adding seven individual factors, one by one (models 2-8), and then summing together the individual benefit of each factor from each model, then the sum of these benefits (+0.027) is not much greater than the combined benefit seen in the single final model that includes all seven factors at once (model 11, +0.022). This suggests limited collinearity (or overlap) between the seven factors, and that each factor each makes a unique contribution to improved discrimination performance.

When grouping these seven factors into two clusters (i.e. four psychosocial factors in model 9; and three biobehavioural factors in model 10), then again the summed improvement in discrimination across both these two models (+0.022) is identical to the discrimination benefit seen in the single final model with all 7 factors (model 11, +0.022). This suggests no collinearity (or overlap) between the two domains of psychosocial factors and biobehavioural factors, with each making complementary (and non-overlapping) contributions to better discrimination performance.

**Supplementary Table 4** Sensitivity analyses, investigating the in C-Statistic from models fitted on pooled data.

Participants: data were pooled across the derivation (Czech Republic, Poland, Russia) and validation (Estonia) countries. Participants with missing data were omitted. There were 19,230 participants with 429 events.

Statistical analysis: Models were fitted as in the main analysis (with initial results shown under section A below). In the absence of external data, internal validation was conducted as follows. Optimism in the C-statistic was estimated using bootstrapping. Specifically, in each bootstrap sample, a new and slightly revised training model was fitted and a C-statistic calculated (results shown in section B); the fitted training model was then applied to the original sample in order to obtain an additional C-statistic (section C). Our estimate of the optimism was the average discrepancy between these two measures (i.e. C minus B, averaging over 1,000 bootstrap samples). Subtracting the estimated optimism from the unadjusted C-statistics (A), results in an optimism-corrected C-statistic (D). Statistical analyses were performed with the R command *validate* (in the package *rms* by Frank Harrell).

Results:


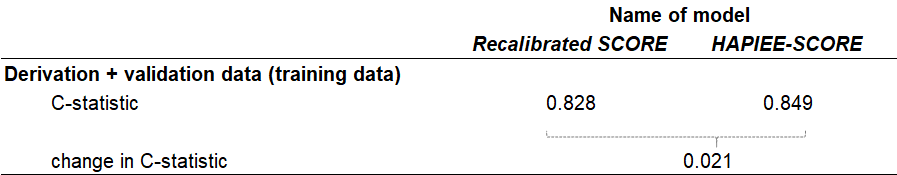

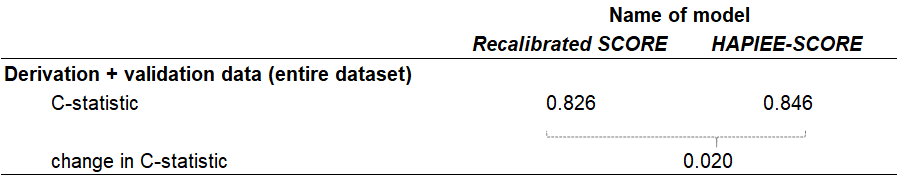
A. Initial model performance on the entire pooled dataset. This found the process of adding new risk factors to increase C-statistic by +0.020. This metric is most comparable to the “change in C-statistic in derivation data”, in table 1 of the main analysis where C-statistic improvement was reported as +0.022.

B. Ordinary bootstrapping was used to identify a random subset of this pooled dataset, where these training models were re-fitted.^[[5]](#footnote-6)^


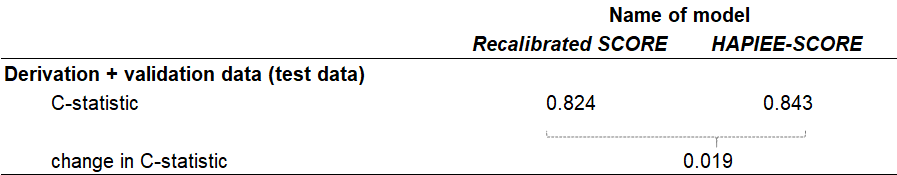
C. When testing the performance of each of the bootstrapped models from B, both models became slightly less accurate, when compared with B. This drop in accuracy (C minus B) is a measure of excess optimism, arising from models in B being overfitted to the data. Optimism in Recalibrated SCORE was 0.003 and in HAPIEE SCORE was 0.006.


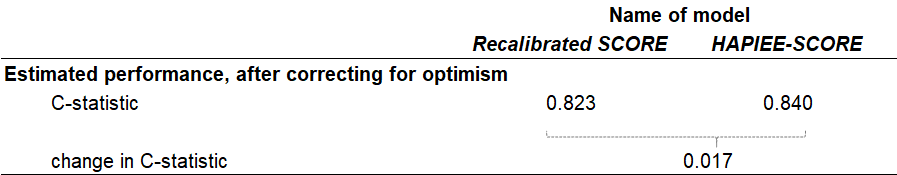
D. The amount of optimism was subtracted from the initial model (A), to estimate likely performance, after accounting for optimism. For Recalibrated SCORE, this was calculated as 0.826-0.003 = 0.823. For HAPIEE SCORE, this was calculated as 0.846-0.006=0.840. Comparing these two optimism-corrected models, suggests that the addition of new risk factors improved C-statistic by +0.017. This metric is most comparable to the “change in C-statistic in validation data”, in table 1 of the main analysis where C-statistic improvement was reported as +0.014.

Conclusions: The addition of new risk factors improved C-statistic in the derivation data by +0.022 (in the main analysis) and +0.020 (in the pooled analysis). When validating model performance, the addition of new risk factors improved C-statistic by +0.014 (in the main analysis, using external validation), and +0.017 (in the pooled analysis, using internal validation). Altogether, this suggests that improvements to discrimination performance are likely to be similar, whether country (Estonia) is used as a to identify the validation data or not.

**Supplementary Figure 5** Reclassification plots. Red circles denote participants who subsequently developed CVD during the follow up, while blue circles denote participants who remained CVD free during follow up. The grey lines denote the 5% absolute risk threshold, above which interventions like statins may be indicated. The perfect model will move red dots into the top left quadrant, while moving blue circles into the bottom right quadrant.

T


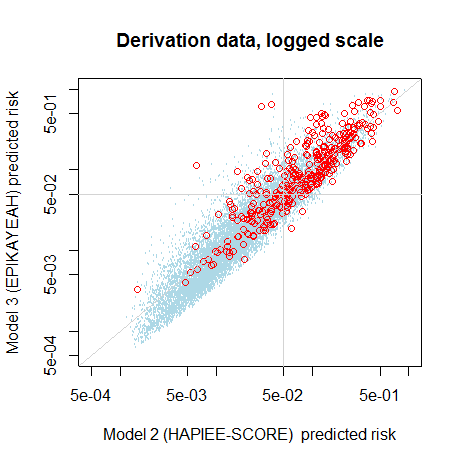

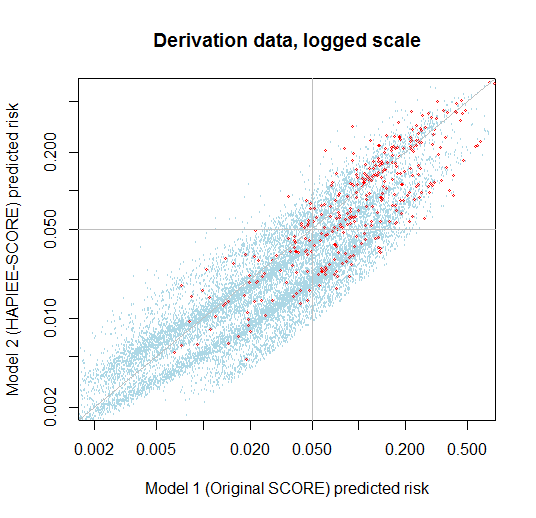

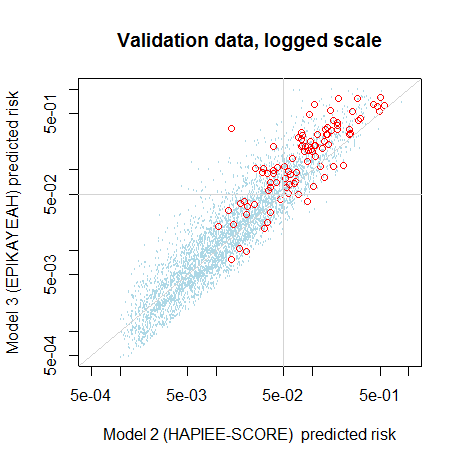

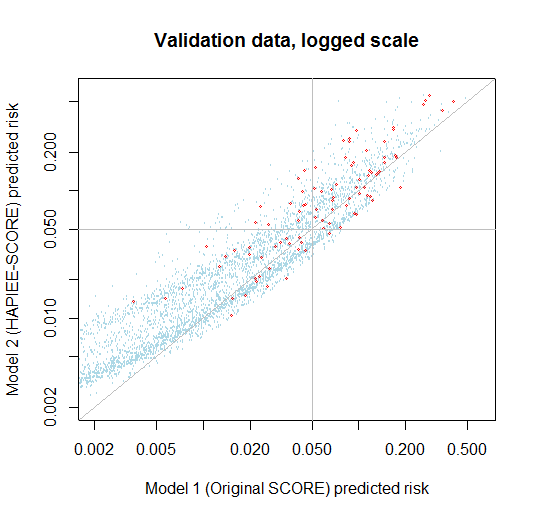


Predicted risk, using the Original SCORE model

Predicted risk, using the Original SCORE model

Predicted risk, using the Recalibrated SCORE model

Predicted risk, using the Recalibrated SCORE model

Predicted risk, using the Recalibrated SCORE model

Predicted risk, using the Recalibrated SCORE model

Predicted risk, using the final HAPIEE SCORE model

Predicted risk, using the final HAPIEE SCORE model

**Supplementary Methods 3 - Multiple imputation - Methods**

Derivation cohort

Between 1% to 13% of the data were assumed to be missing at random for the following variables: depression, HDL cholesterol, total cholesterol, Systolic Blood Pressure, BMI:


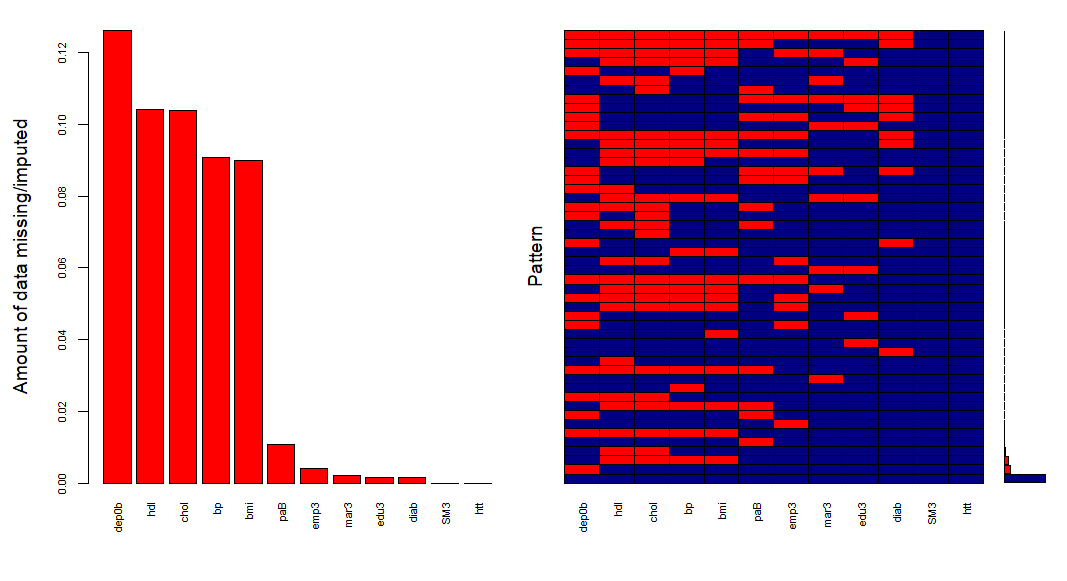


20 datasets were generated that imputed missing data for these five variables (plus for six variables where the degree of missingness was <1%), using the package mice (seed=12345). The predictor matrix was defined automatically using the *quickpred* command, with the following five variables with no missing forced into the predictor matrix: age, gender, country, cvd event at end of follow up, duration of follow-up.

The density plots of the resulting imputed variables are shown on the below.

(Red line = one of the 20 imputed datasets. Blue line= complete case data)

The imputed derivation cohort had 19’043 participants followed up for a median time of 7.2 years, to identify 509 events.


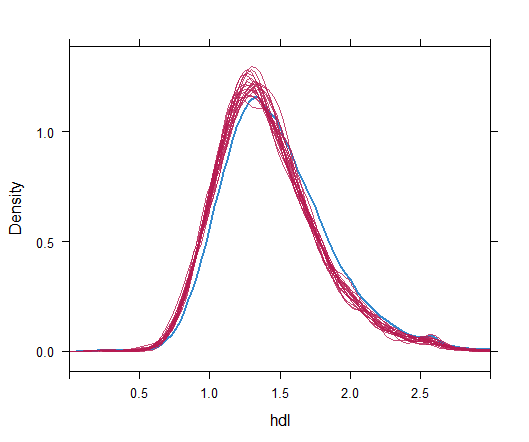

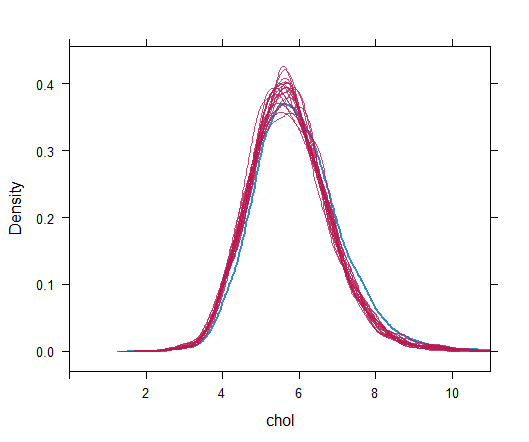

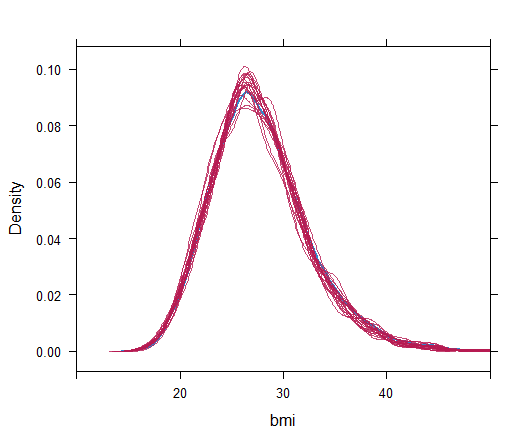

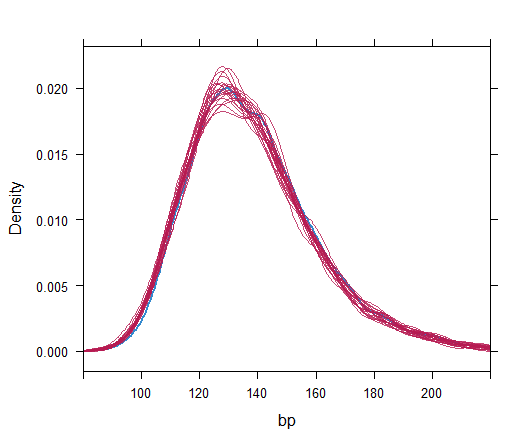


Validation cohort

78% of the data for total cholesterol and HDL cholesterol were assumed to be missing (as random individuals were picked for subsequent cholesterol measurement from frozen sample.)

Missing at random was assumed for the following variables: 19% of the data for Physical inactivity, 6% for employment status, 4% for marital status. The remaining five variables were missing in <1% of the participants:


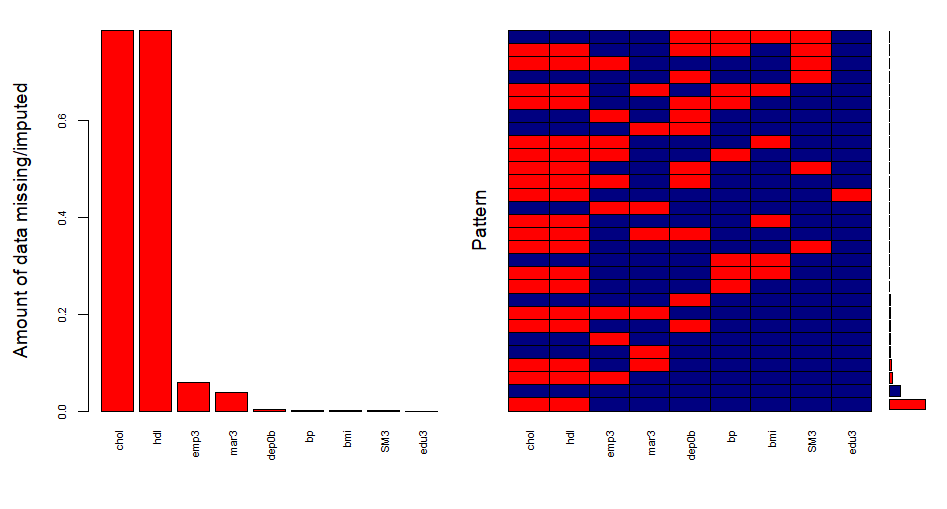


20 datasets were generated that imputed missing data for these 12 variables, using the package mice (seed=12345). The predictor matrix was defined automatically using the quickpred command, with the following five variables with no missing forced into the predictor matrix: age, gender, country, cvd event at end of follow up, duration of follow-up.

Density plots of the resulting imputed variables are shown below.

(Red line = one of the 20 imputed datasets. Blue line= complete case data):

The imputed validation cohort had 23’233 participants followed up for a median time of 7.6 years, to identify 431 events.


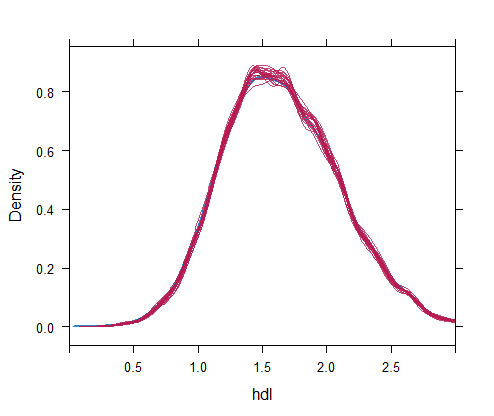

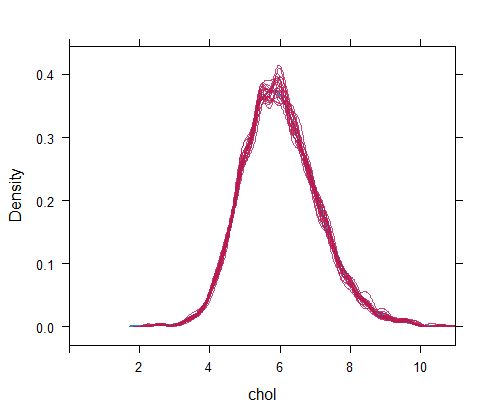


**Multiple imputation – Results**

We report below the same findings as quoted in the abstract of the main paper (which used a complete case dataset), however this time using data from the first imputed dataset:

*“Discrimination of the original SCORE model (C-statistic 0.78 in the derivation and 0.83 in the validation cohorts) was improved in Recalibrated SCORE (0.81 and 0.85) and HAPIEE SCORE models (0.83 and 0.86).*

*After dichotomizing risk at the clinically meaningful threshold of 5%, and when comparing the final HAPIEE SCORE model against the original SCORE model, the Net Reclassification Improvement (NRI) was 0.15 (95% CI: 0.11, 0.19) in the derivation and 0.05 (95% CI: 0.02, 0.09) in the validation cohort.”*

Detailed results are shown in the Supplementary Tables 5-7 below:

**Supplementary Table 5** - Discrimination performance of three cardiovascular prediction models, as measured by Harrell’s C-statistic, using imputed data instead of complete case data.


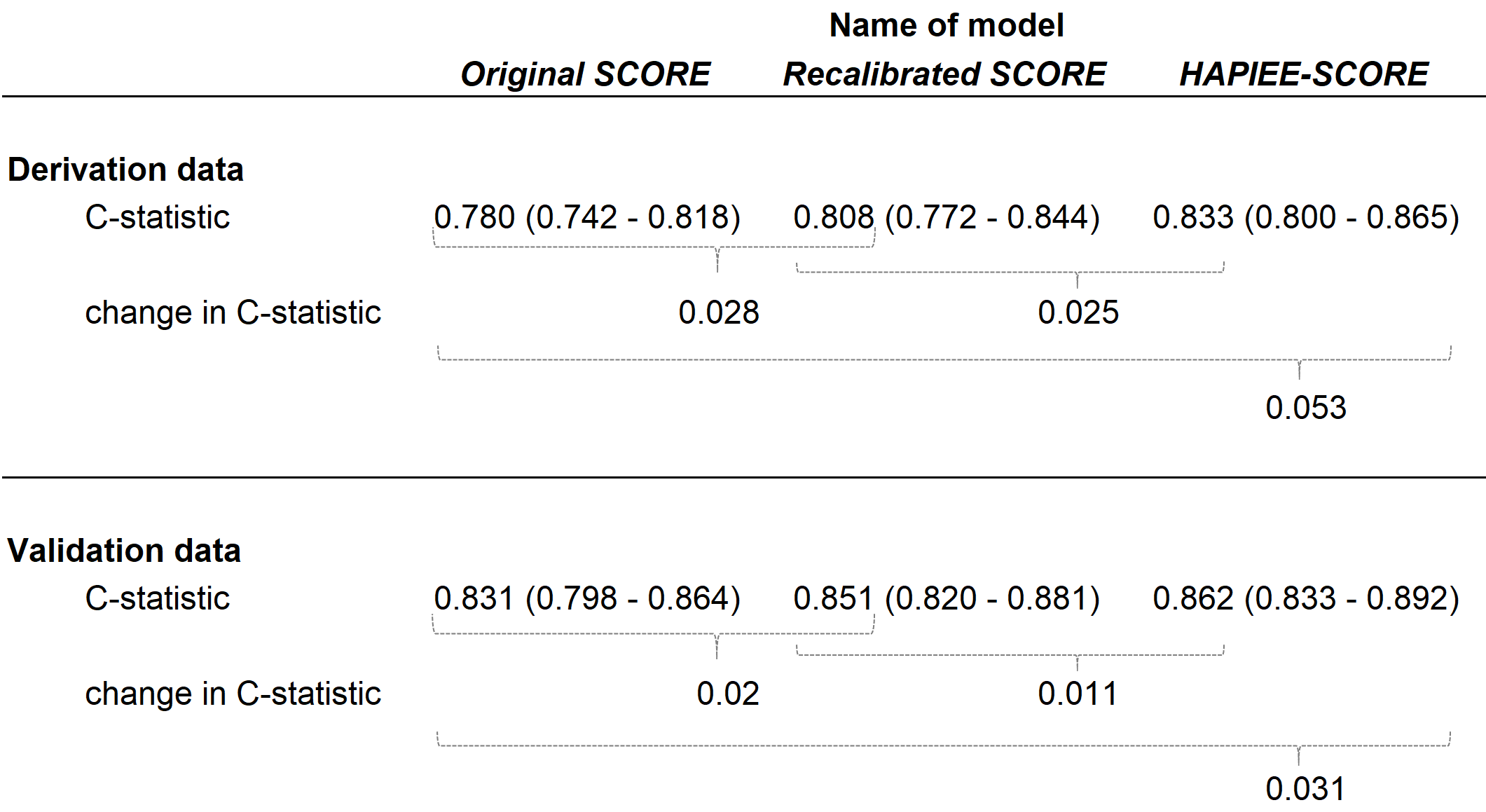


**Supplementary Table 6** Changes to reclassification across three cardiovascular prediction models, in the derivation cohort, using imputed data instead of complete case data.


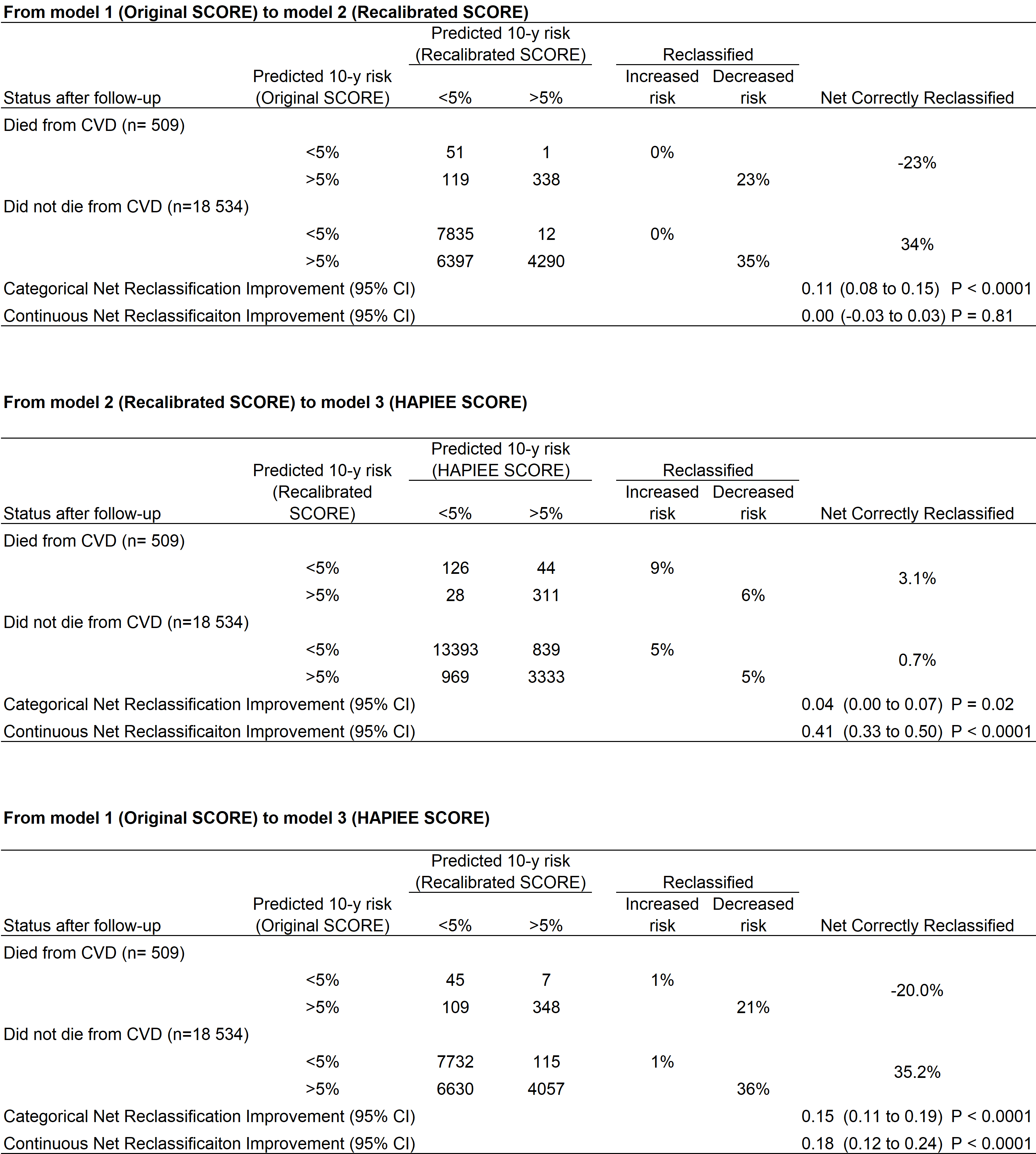


**Supplementary Table 7** Changes to reclassification across three cardiovascular prediction models, in the validation cohort, using imputed data instead of complete case data.


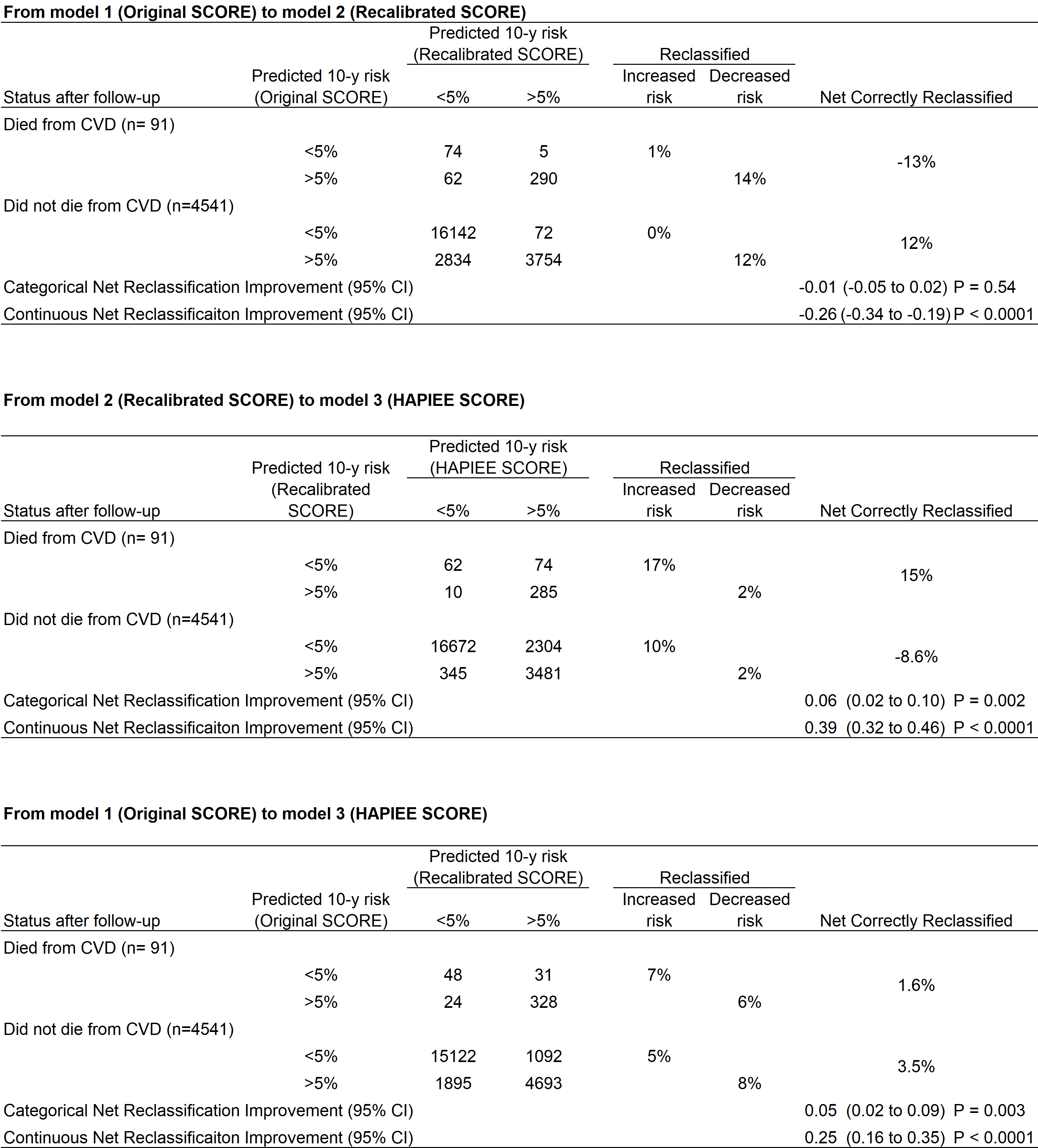


1. Vittinghoff E, McCulloch CE. Relaxing the rule of ten events per variable in logistic and cox regression. *Am J Epidemiol* 2007;165(6):710–8. [↑](#footnote-ref-2)
2. Harrell F. Internet Statistical Forum “Cross Validated”. 2014. <https://stats.stackexchange.com/questions/104518/calculate-a-95-confidence-interval-and-p-value-for-the-change-in-c-statistic-us> [↑](#footnote-ref-3)
3. Pencina MJ, D'Agostino Sr RB, D'Agostino Jr RB, Vasan RS. Evaluating the added predictive ability of a new marker: from area under the ROC curve to reclassification and beyond. *Stat In Med* 2008;27(2):157-72. [↑](#footnote-ref-4)
4. Wang H, Naghavi M, Allen C, Barber RM, Bhutta ZA, Carter A, Casey DC, Charlson FJ, Chen AZ, Coates MM, Coggeshall M. Global, regional, and national life expectancy, all-cause mortality, and cause-specific mortality for 249 causes of death, 1980–2015: a systematic analysis for the Global Burden of Disease Study 2015. *Lancet* 2016;388(10053):1459-544. [↑](#footnote-ref-5)
5. Efron B, Tibshirani R. Improvements on cross-validation: The .632+ bootstrap method. *JASA* 1997;92:548–560. Eq 2.10 [↑](#footnote-ref-6)
